# Mechanisms and therapeutic prospects of DNA methylation–mucosal innate immunity crosstalk in inflammatory bowel disease

**DOI:** 10.3389/fimmu.2026.1877804

**Published:** 2026-06-09

**Authors:** Tao Zhang, Zhetan Ren, Zhanshuo Kang, Zhengchao Pan, Gang Wei, Ling Wang, Ru Man, Jirun Peng, Yongduo Yu

**Affiliations:** 1 Liaoning University of Traditional Chinese Medicine, Shenyang, China; 2 Second Affiliated Hospital, Liaoning University of Traditional Chinese Medicine, Shenyang, China; 3 Department of General Surgery, Beijing Shijitan Hospital, Capital Medical University, Beijing, China; 4 Ninth School of Clinical Medicine, Peking University, Beijing, China

**Keywords:** DNA methylation, epigenetic regulation, inflammatory bowel disease, innate immunity, intestinal mucosal barrier, mucosal immunity

## Abstract

Inflammatory bowel disease (IBD) comprises a group of chronic and relapsing intestinal inflammatory disorders whose pathogenesis and progression are closely associated with disruption of the intestinal mucosal barrier, dysregulated immune responses, and altered epigenetic regulation. The innate immune system is a central component of mucosal host defense and plays a pivotal role in pathogen recognition, inflammatory signal transduction, immune-cell functional regulation, and maintenance of barrier homeostasis. In recent years, DNA methylation has been increasingly recognized as an important mechanism contributing to the development and persistence of innate immune dysregulation in IBD by modulating the transcriptional activity of immune-related genes, inflammatory pathway genes, and barrier-function genes. Conversely, persistently activated innate immune responses may reshape DNA methylation patterns through inflammatory cytokines, oxidative stress, and signaling pathways such as NF-κB and JAK/STAT, thereby forming a dynamic bidirectional regulatory network. This review systematically summarizes the mechanisms underlying the crosstalk between DNA methylation and the innate immune system in IBD, with particular emphasis on its potential roles in inflammatory initiation, immune-cell infiltration, stabilization of pro-inflammatory phenotypes, mucosal barrier injury, inflammatory memory, and disease relapse. We further propose a conceptual framework termed the “DNA methylation–innate immunity interaction axis.” Current evidence suggests that this interaction axis may provide a new mechanistic perspective for understanding the maintenance of chronic inflammation and recurrent disease activity in IBD. It may also offer a theoretical basis for combined epigenetic–immune interventions, biomarker development, and optimization of precision therapeutic strategies. Future studies integrating single-cell omics, spatial omics, longitudinal cohorts, and functional validation are warranted to further define the cell-type specificity, stage-dependent effects, and clinical translational potential of this axis.

## 1 Introduction

Inflammatory bowel disease (IBD) is a group of immune-related disorders characterized primarily by chronic and relapsing intestinal inflammation, mainly including Crohn’s disease (CD) and ulcerative colitis (UC) (1). Patients commonly present with abdominal pain, diarrhea, mucus- and pus-containing bloody stools, weight loss, and extraintestinal manifestations. Recurrent disease flares can markedly impair quality of life and impose a substantial long-term healthcare burden (2). Although the etiology of IBD has not yet been fully elucidated, current evidence generally indicates that its onset and progression result from the combined effects of multiple factors, including genetic susceptibility, environmental exposures, intestinal mucosal barrier disruption, dysregulated immune responses, and altered epigenetic regulation (3).

The innate immune system is a core component of the intestinal mucosal defense network and plays a fundamental role in recognizing microbe-associated molecular patterns, maintaining barrier homeostasis, and initiating inflammatory responses (4). Under physiological conditions, the intestinal innate immune system maintains a dynamic balance between eliminating harmful stimuli and preserving mucosal immune tolerance (5). In IBD, however, the functional state of innate immune-related cells becomes dysregulated, thereby contributing to the initiation, amplification, and persistence of chronic intestinal inflammation (6). In recent years, DNA methylation, as a major form of epigenetic regulation, has attracted increasing attention in the context of IBD (7). By modulating transcriptional activity at gene promoter or enhancer regions, DNA methylation can influence the expression of immune-related genes, barrier-function genes, and inflammatory pathway-associated genes, thereby linking environmental stimuli, epigenetic alterations, and immune dysregulation (8, 9).

Emerging evidence suggests that DNA methylation and innate immunity may form a dynamic bidirectional regulatory network (10). On the one hand, aberrant DNA methylation can reshape the transcriptional programs of innate immune cells, altering the magnitude of inflammatory responses and their functional phenotypes (11). On the other hand, inflammatory cytokines and reactive oxygen species generated during innate immune activation, together with signaling pathways such as NF-κB and JAK/STAT, may further regulate the expression and activity of DNA methyltransferases (DNMTs) and demethylases such as ten-eleven translocation enzymes (TETs). These processes may induce further methylation reprogramming and establish a potential positive-feedback regulatory loop (12).

**Figure 1:**
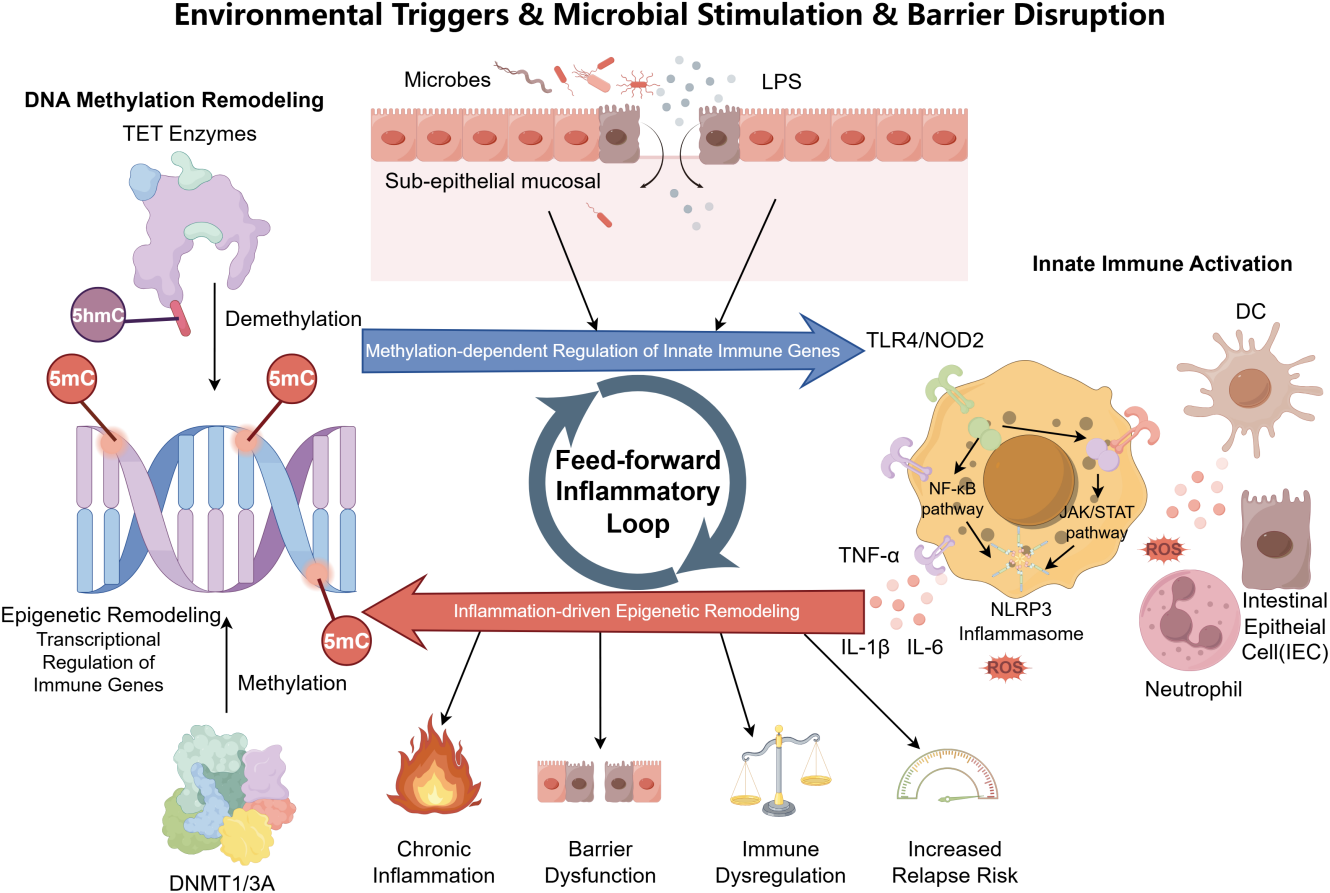
Schematic overview of the DNA methylation–innate immunity crosstalk axis in inflammatory bowel disease. Environmental triggers, microbial stimulation, LPS exposure, and intestinal barrier disruption facilitate the translocation of microbial products into the subepithelial mucosal compartment, thereby activating innate immune cells, including macrophages, dendritic cells, neutrophils, and intestinal epithelial cells. Activation of TLR4/NOD2, NF-κB, JAK/STAT, and NLRP3 inflammasome signaling promotes the release of TNF-α, IL-1β, IL-6, and reactive oxygen species (ROS), amplifying mucosal inflammation. In turn, inflammatory mediators and oxidative stress may influence DNMT1/3A, TET enzymes, and the dynamic balance between 5mC and 5hmC, leading to DNA methylation remodeling and altered transcriptional regulation of immune-related genes. This immune–epigenetic interaction may establish a feed-forward inflammatory loop that contributes to chronic inflammation, barrier dysfunction, immune dysregulation, and increased relapse risk.

However, current studies have not yet sufficiently integrated the bidirectional regulatory relationship between DNA methylation and innate immunity in a systematic manner. Therefore, focusing on the mutual regulation between DNA methylation and innate immunity, this review systematically discusses the mechanisms by which these two processes contribute to the onset and progression of IBD, and further analyzes their potential applications as therapeutic targets and biomarkers. In this review, we propose the conceptual framework of a “DNA methylation–innate immunity interaction axis.” Rather than representing a single linear signaling pathway, this axis constitutes a multilayered interactive network orchestrated by DNA methylation dynamics, innate immune recognition, inflammatory signal transduction, and the regulation of epigenetic enzymes, as illustrated in **Figure 1**.

## 2 DNA methylation abnormalities and epigenetic regulatory features in IBD

During the onset and progression of IBD, epigenetic regulation is regarded as an important link between environmental factors and altered gene expression (13). In recent years, the role of epigenetic regulation in IBD has gradually shifted from a supplementary explanation to a major focus of mechanistic research (14). Among various epigenetic mechanisms, DNA methylation is one of the most classical forms of epigenetic modification and occupies a central position in transcriptional regulatory networks (15). With the development of high-throughput sequencing and multi-omics technologies, IBD-associated DNA methylation alterations have been increasingly mapped in a systematic manner, and the scope of research has expanded from individual inflammation-related genes to broader regulatory networks (16).

DNA methylation refers to the covalent addition of a methyl group to cytosine residues in DNA, predominantly at CpG dinucleotide sites (17). This process is mainly mediated by DNA methyltransferases (DNMTs), whereas DNA demethylation is closely associated with the ten-eleven translocation (TET) family of enzymes (18). Methylation status can alter gene transcriptional activity by influencing chromatin conformation and transcription factor binding (19).

In gene promoter regions, higher levels of methylation are generally associated with transcriptional repression, whereas reduced methylation is more permissive to gene expression (20). However, this regulatory relationship is not fixed, but varies according to genomic region and cell type. DNA methylation is also dynamically plastic, and its status may be influenced by inflammatory stimuli, oxidative stress, and changes in the metabolic environment. It therefore serves as an important mediator through which environmental factors regulate gene expression (21).

Current studies have shown widespread DNA methylation alterations in both intestinal mucosal tissues and peripheral blood cells from patients with IBD (22). These alterations are characterized by the coexistence of hypermethylation and hypomethylation at multiple loci, involving a range of biological processes such as inflammatory responses, epithelial barrier function, and cellular stress (23). Overall, these changes suggest broad remodeling of transcriptional regulatory networks in IBD.

At the level of specific genes, hypomethylation in the promoter regions of certain inflammation-related genes is consistent with their increased expression, whereas some genes involved in barrier maintenance and immune regulation tend to exhibit increased methylation (24). In addition, signal transduction pathways are also affected by DNA methylation-mediated regulation. For example, changes in the methylation status of molecules related to the NF-κB signaling pathway and pattern-recognition receptor-associated genes are closely linked to adjustments in transcriptional activity (25).

Different studies have also indicated that DNA methylation patterns may vary according to tissue origin, disease subtype, and stage of inflammatory activity (26). For instance, varying degrees of methylation differences have been observed between the colon and ileum, between CD and UC, and between active and remission stages (27). These differences suggest a potential association between DNA methylation and disease phenotype, although the specific functional pathways involved remain incompletely defined. Meanwhile, technical differences arising from different detection platforms and analytical strategies further contribute to heterogeneity among study findings.

Altered DNA methylation may participate in IBD-related pathological processes by regulating the expression of multiple classes of genes (28). Changes in the methylation status of cytokine-related genes are associated with the intensity of local inflammation (29). Methylation alterations in epithelial cell-related genes may affect mucosal barrier structure and permeability, thereby reshaping the intestinal luminal microenvironment (30). In addition, regulatory changes in genes involved in cellular stress responses and tissue repair may contribute to tissue injury and repair during persistent disease activity (31).

At the clinical level, DNA methylation also holds potential application value. Certain methylation loci have been used to assess disease activity or distinguish different disease phenotypes (32). Given the partial reversibility of DNA methylation, its potential as an interventional target has also attracted attention. However, current studies remain largely exploratory, and systematic evaluations of specific methylation loci and intervention strategies are still limited.

## 3 Molecular mechanisms of DNA methylation–innate immunity crosstalk

During the development and progression of inflammatory bowel disease, altered DNA methylation and innate immune dysregulation often coexist, but the mechanistic links between the two have not yet been fully clarified. Existing evidence suggests that DNA methylation is not only an important mechanism of gene-expression regulation, but may also participate in the modulation of innate immune responses at multiple levels, including pathogen recognition, inflammatory signal transduction, and amplification of inflammatory effector responses (33, 34). At the same time, a persistent inflammatory microenvironment may, in turn, reshape epigenetic states, thereby forming a dynamic regulatory relationship between DNA methylation and innate immunity (35).

### 3.1 Molecular mechanisms by which DNA methylation regulates innate immunity

DNA methylation may participate in the initiation, maintenance, and amplification of innate immune responses by regulating the transcriptional activity of key genes (36). To systematically present the relevant molecular mechanisms, **Table 1** summarizes DNA methylation-related targets, major effector cells, and immune effects across different regulatory levels. Current evidence is mainly derived from analyses of intestinal mucosal tissues, *in vitro* experiments, and animal models, and differences remain among studies regarding the direction and magnitude of specific molecular changes.

**Table 1:** Regulatory layers, representative targets, and immune effects of DNA methylation-mediated innate immune modulation in IBD.

Regulatory level	Methylation-related target	Target cell	Mechanism of action and immune effect	References
Pathogen-recognition level	TLR4, NOD2	Macrophages, dendritic cells	Enhanced expression of TLR/NOD receptors, increased sensitivity to microbial signal recognition, and initiation of pro-inflammatory responses	(37, 38)
Pathogen-recognition level	CD14	Macrophages	Enhanced LPS-binding capacity and increased activation of inflammatory signaling	(39, 40)
Inflammatory signaling level	NF-κB-related genes	Multiple innate immune cells	Enhanced activation of the NF-κB pathway and sustained release of inflammatory cytokines, including TNF-α, IL-1β, and IL-6	(41, 42)
Inflammatory signaling level	JAK/STAT pathway genes	Macrophages, dendritic cells	Enhanced cytokine signal transduction and sustained upregulation of inflammatory cytokine expression	(43, 44)
Inflammasome level	NLRP3	Macrophages	Enhanced inflammasome activation and increased IL-1β release	(45, 46)
Inflammasome level	CASP1	Macrophages	Increased caspase-1 activity and enhanced maturation and release of inflammatory mediators	(47, 48)
Cell-function level	iNOS, ARG1	Macrophages	Shift of macrophages toward a pro-inflammatory phenotype and amplification of inflammatory responses	(49, 50)
Cell-function level	IL-12, IL-10	Dendritic cells	Altered cytokine secretion profile and modulation of the intensity and direction of local immune responses	(51, 52)
Cellular-response level	CXCL8 (IL-8)	Neutrophils	Enhanced chemotactic signaling and increased neutrophil recruitment	(53, 54)
Barrier-immunity level	ZO-1, Occludin	Epithelial cells	Disruption of tight junction structure and increased epithelial permeability	(55, 56)
Barrier-immunity level	MUC2	Epithelial cells	Reduced mucus secretion and impaired mucosal barrier function	(57, 58)
Stress-related regulation	HIF-1α	Epithelial cells, macrophages	Enhanced hypoxia-associated inflammatory responses and maintenance of immune-cell activity	(59, 60)

**Figure 2:**
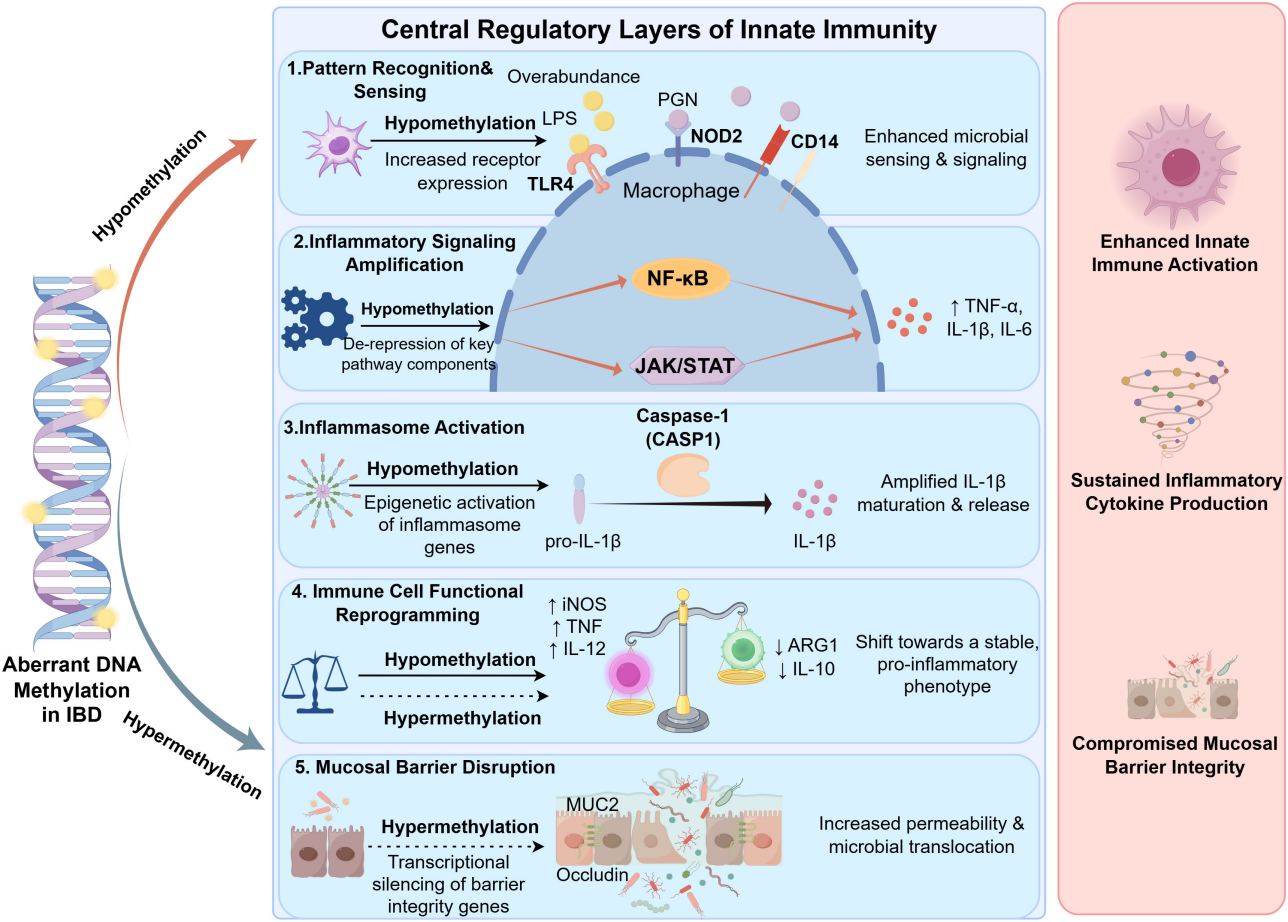
Major regulatory layers through which DNA methylation modulates innate immune responses in IBD. Aberrant DNA methylation in IBD may reshape innate immune responses by altering the transcriptional status of immune- and barrier-related genes. Hypomethylation of pattern-recognition receptor genes, such as TLR4, NOD2, and CD14, may enhance microbial sensing in response to LPS, PGN, and other microbial products. Epigenetic de-repression of NF-κB and JAK/STAT pathway components may promote TNF-α, IL-1β, and IL-6 production, while activation of inflammasome-related genes may facilitate CASP1-mediated IL-1β maturation and release. Hypomethylation of pro-inflammatory genes and hypermethylation of anti-inflammatory genes may shift immune cells toward a stable pro-inflammatory phenotype. Hypermethylation of barrier-related genes, including MUC2 and Occludin, may impair barrier integrity, increase permeability and microbial translocation, and further enhance innate immune activation. Solid arrows indicate activating effects, whereas dashed lines indicate inhibitory or suppressive effects. DNMT1/3A, DNA methyltransferase 1/3A; TET, ten-eleven translocation enzymes; DC, dendritic cell.

Overall, these epigenetic alterations mainly contribute to the remodeling of innate immune responses by affecting pathogen recognition, inflammatory signal transduction, amplification of inflammatory effector responses, and regulation of barrier function. The regulation of innate immunity by DNA methylation can be summarized across several interconnected levels, including pathogen recognition and sensing, inflammatory signal amplification, inflammasome activation, immune-cell functional reprogramming, and mucosal barrier disruption (**Figure 2**).

At the levels of pathogen recognition and signal transduction, DNA methylation may influence the responsiveness of innate immune cells to microbial signals by regulating the expression of pattern-recognition receptor-related genes (61). In the intestinal mucosa of patients with IBD, several studies have observed that reduced methylation levels in TLR4-related regions are consistent with its increased expression, a change that corresponds to enhanced lipopolysaccharide responsiveness and increased release of inflammatory cytokines (62). *In vitro* experiments further indicate that modulation of TLR4 methylation status can alter the magnitude of macrophage responses to microbial stimulation (63). In downstream signaling pathways, methylation changes in genes associated with the NF-κB signaling pathway correspond to altered transcriptional activity. Animal studies have shown that intervention in relevant epigenetic states can affect the expression levels of inflammatory cytokines (64). Similar observations have also been reported for the JAK/STAT pathway, in which methylation alterations in related genes are associated with enhanced cytokine signaling responses, although findings remain inconsistent across studies (65).

At the level of immune-cell function, DNA methylation may contribute to the functional remodeling of innate immune cells, including macrophages, dendritic cells, and neutrophils (66). During macrophage polarization, changes in the expression of key genes such as iNOS and ARG1 correspond to the inflammatory state, and some studies suggest that their transcriptional regulation may be influenced by alterations in local methylation patterns (67). Cellular experiments have shown that intervention in relevant epigenetic states can affect the shift of macrophages toward a pro-inflammatory phenotype, thereby modifying the intensity of inflammatory responses (68). In dendritic cells, the expression levels of IL-12 and IL-10 may vary under different inflammatory conditions, consistent with changes in their immunoregulatory functions (69). In addition, the transcriptional regulation of neutrophil chemoattractants such as CXCL8 is closely associated with inflammatory-cell recruitment, and their altered expression may be influenced by changes in local methylation status (70).

Inflammasomes represent key nodes in the amplification of innate immune effector responses, and the expression of inflammasome-related molecules is also affected by epigenetic regulation. NLRP3 and CASP1 may be aberrantly expressed in IBD tissues, and their expression levels are associated with inflammatory disease activity (71). *In vitro* studies have shown that modulation of the methylation status of related genes can influence the degree of inflammasome activation, accompanied by changes in IL-1β release (72). Animal models also suggest that the extent of inflammasome activation is associated with the severity of intestinal inflammation. However, findings vary under different experimental conditions, and the specific regulatory mechanisms require further clarification (73).

Beyond immune cells, altered DNA methylation may also participate in the remodeling of the local immune microenvironment by affecting the expression of epithelial barrier-related genes (74). In patients with IBD, reduced expression of tight junction-related genes, such as ZO-1 and Occludin, corresponds to impaired barrier integrity, and some studies suggest that this change may be associated with altered methylation levels (75). Similarly, abnormal MUC2 expression is associated with reduced mucus barrier function. Damage to the barrier structure may facilitate the entry of microbe-associated molecules into the mucosal layer, thereby enhancing stimulation of the innate immune system (76). In addition, changes in HIF-1α expression under inflammatory and hypoxic conditions are associated with the maintenance of the local inflammatory microenvironment, and its regulation may contribute to the sustained adjustment of immune-cell functional states (77).

### 3.2 Regulation of DNA methylation by innate immunity

The innate immune system can also reciprocally influence epigenetic regulatory states and participate in the modulation of DNA methylation patterns (78). Accumulating evidence suggests that chronic activation of inflammatory cytokine signaling, oxidative stress, and key transcriptional pathways can alter CpG methylation levels by regulating the expression of DNA methyltransferases (DNMTs) and demethylation-associated enzymes, including the ten-eleven translocation (TET) family, thereby further reshaping the transcriptional landscape of immune-related genes (79). At the cytokine level, *in vitro* studies have shown that TNF-α and IL-6 can induce changes in DNMT1 or DNMT3A expression, accompanied by altered methylation states within specific promoter regions (80, 81). Such alterations may contribute to the regulation of inflammation-associated gene expression and promote the establishment of relatively stable pro-inflammatory transcriptional programs in immune cells under persistent stimulation (82). Importantly, the effects of inflammatory cytokines on DNA methylation appear to be highly dependent on cell type, stimulus intensity, target gene function, and local chromatin context (83). These variables may explain the discrepancies observed among studies regarding specific target genes and regulatory directions, while also highlighting the pronounced site specificity and context dependency of innate immune signaling–mediated DNA methylation regulation.

Beyond cytokine signaling, oxidative stress represents another critical link connecting immune responses and epigenetic regulation. Reactive oxygen species (ROS), which are abundantly generated during inflammation, can participate in DNA demethylation processes through modulation of TET enzyme activity (84, 85). Experimental studies in animal models have demonstrated that elevated ROS levels are associated with alterations in 5-hydroxymethylcytosine (5hmC) abundance, suggesting a potential role for ROS in transcriptional regulation through the modulation of DNA demethylation dynamics (86). In parallel, oxidative stress may also indirectly affect DNMT activity, thereby disturbing the dynamic equilibrium between methylation and demethylation (87). Notably, the epigenetic effects of ROS are likely influenced by their intensity, duration, and spatial distribution. Transient or moderate ROS elevation appears more closely associated with reversible TET-dependent demethylation, whereas sustained high ROS levels may induce oxidative DNA damage, disrupt DNMT/TET balance, and promote aberrant stabilization of methylation patterns. These observations raise the possibility that a relatively permissive window for ROS-mediated epigenetic regulation may exist within chronic IBD lesions, although its precise boundaries remain to be clarified.

At the signaling pathway level, persistently activated inflammatory pathways are increasingly recognized as central nodes linking inflammatory responses to epigenetic reprogramming. Following inflammatory stimulation, activated NF-κB p65 translocates into the nucleus and binds to promoter or enhancer regions of target genes (88). In addition, p65 can interact with co-activators and co-repressors to influence the recruitment of DNMT1, DNMT3A, or TET family enzymes to specific genomic loci (89). At pro-inflammatory gene loci, p65-associated transcriptional complexes may promote chromatin accessibility and transcriptional activation, frequently accompanied by localized hypomethylation (90). Conversely, at certain barrier-protective or immunoregulatory gene loci, the inflammatory microenvironment may facilitate the enrichment of DNMT-associated complexes, resulting in promoter hypermethylation and transcriptional suppression (91). Meanwhile, NF-κB activation may further modulate TET family enzyme expression through the IL-6/STAT3 axis, thereby generating differential patterns of DNMT-mediated methylation and TET-mediated demethylation across distinct genomic regions (92). Such locus-specific regulation may help explain why both hypomethylation and hypermethylation can coexist within the same inflammatory milieu, although the decoding molecules and cofactor networks responsible for selective targeting remain largely unresolved.

In addition, signaling pathways such as JAK/STAT may participate in similar regulatory processes during inflammatory signal transduction. Cytokines including IL-6 can activate transcription factors such as STAT3 through the JAK/STAT pathway, thereby influencing inflammatory gene transcription and the recruitment of epigenetic enzymes (93). STAT3-associated transcriptional complexes may couple cytokine signaling to methylation or demethylation remodeling at specific genomic regions through interactions with DNMTs or TETs (94). Therefore, the key determinant of innate immune signaling–mediated DNA methylation regulation may not solely lie in altered DNMT/TET expression levels, but rather in how these enzymes are selectively recruited to target genomic loci under the coordinated guidance of NF-κB, JAK/STAT signaling, and associated cofactor networks.

### 3.3 Regulatory features of DNA methylation–innate immunity crosstalk

The relationship between DNA methylation and innate immunity represents a multilayered regulatory network characterized by bidirectional feedback interactions. Within this network, pattern-recognition receptors, inflammatory signaling pathways, epigenetic regulatory enzymes, and inflammation- and barrier-related target genes collectively constitute the principal nodes of the interaction axis (95). Among these components, pattern-recognition receptors such as TLR4, NOD2, and CD14 function at the forefront of microbial signal sensing, whereas pathways involving NF-κB, JAK/STAT, the NLRP3 inflammasome, and ROS signaling connect immune activation to inflammatory amplification. Meanwhile, epigenetic regulators including DNMT1, DNMT3A, and TET family enzymes further modulate the transcriptional states of inflammation- and barrier-associated genes, such as TNF, IL1B, CXCL8, MUC2, ZO-1, and Occludin (96).

During this regulatory process, aberrant DNA methylation may initially alter the response threshold of innate immune cells to microbial stimuli. For example, hypomethylation within TLR4-related regions can promote TLR4 upregulation, rendering macrophages and dendritic cells more sensitive to lipopolysaccharide (LPS) and related microbial signals, thereby further enhancing NF-κB-mediated inflammatory signaling (97). Subsequent NF-κB activation promotes the release of inflammatory cytokines such as TNF-α and IL-1β, which may in turn reshape the methylation landscape of inflammation- and barrier-related genes through modulation of DNMT and TET expression or activity (98). Concurrently, aberrant methylation of barrier-associated genes, including MUC2, ZO-1, and Occludin, may impair mucus layer integrity and tight junction architecture, facilitating the translocation of microbial products into the mucosal compartment and thereby further amplifying pattern-recognition receptor–mediated immune activation (99).

**Figure 3:**
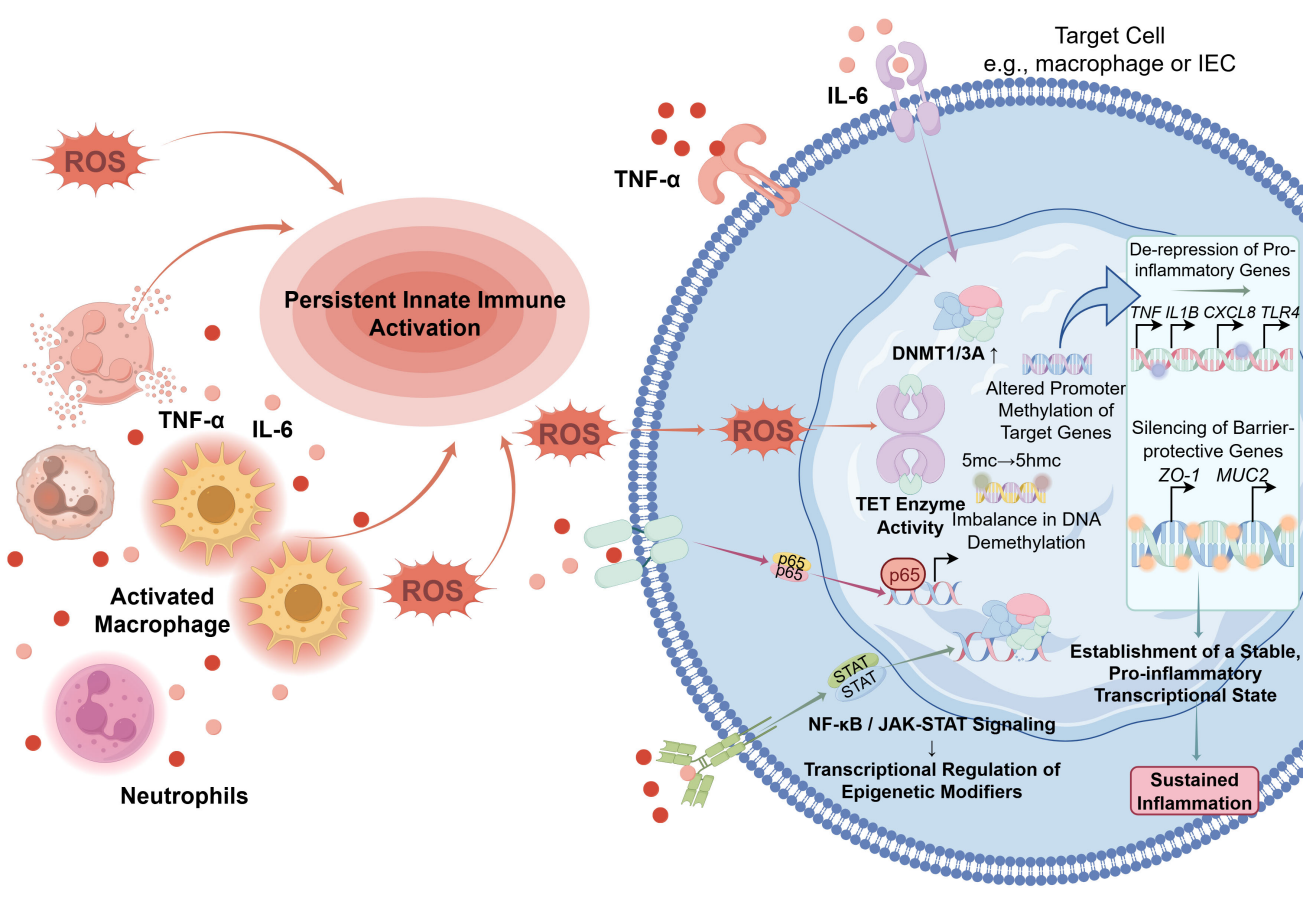
Persistent innate immune activation drives DNA methylation remodeling and stabilization of a pro-inflammatory transcriptional state. Persistently activated innate immune cells, including macrophages and neutrophils, release TNF-α, IL-6, and ROS, which act on target cells such as macrophages and intestinal epithelial cells. These inflammatory signals may regulate DNMT1/3A, TET activity, and the 5mC/5hmC balance, thereby altering promoter methylation of target genes. De-repression of pro-inflammatory genes, including TNF, IL1B, CXCL8, and TLR4, promotes sustained inflammation, whereas silencing of barrier-protective genes such as ZO-1 and MUC2 may aggravate barrier dysfunction. Together, these changes contribute to the establishment of a stable pro-inflammatory transcriptional state.

Collectively, these findings suggest that aberrant DNA methylation and innate immune activation in IBD form a continuous feedback loop characterized by “epigenetic alteration–immune signal amplification–inflammatory microenvironment remodeling–secondary epigenetic reprogramming.” Crosstalk among distinct regulatory nodes may additionally occur through shared cytokine networks, oxidative stress signaling, and inflammasome pathways, gradually shifting local inflammation from transient activation toward persistent maintenance (**Figure 3**) (100). From this perspective, the DNA methylation–innate immunity interaction axis may be more appropriately conceptualized as a dynamic and network-oriented epigenetic–immune regulatory framework.

In addition, dysregulated adaptive immunity may also participate in the modulation of the DNA methylation–innate immunity interaction axis. Imbalances between Th17 and Treg cells are closely associated with the persistence of chronic mucosal inflammation (101). Epigenetically reprogrammed dendritic cells may further influence naïve T-cell differentiation toward Th17 or Treg phenotypes by altering antigen-presenting capacity, co-stimulatory molecule expression, and the secretion profiles of cytokines such as IL-6, IL-1β, IL-23, and IL-10 (102, 103). These findings indicate that epigenetically remodeled innate immune cells not only contribute to the initiation and amplification of inflammation, but may also indirectly sustain chronic inflammatory responses through their immunoregulatory and immune-polarizing functions.

## 4 Dynamic roles of DNA methylation–innate immunity crosstalk in the onset and progression of IBD

The development and progression of inflammatory bowel disease (IBD) result from the persistent interplay between immune responses and epigenetic regulation across different disease stages (104). Throughout disease evolution, the relationship between DNA methylation and the innate immune system exhibits dynamic and stage-dependent characteristics. Distinct regulatory patterns emerge during the initiation of immune activation, inflammatory expansion, chronic maintenance, and disease relapse (105). Accordingly, examining the DNA methylation–innate immunity interaction from the perspective of disease progression may provide a more comprehensive understanding of both IBD pathogenesis and its sustained evolution.

### 4.1 Initiation stage of IBD

#### 4.1.1 Aberrant activation of innate immunity

Under physiological intestinal conditions, innate immune cells respond to microbial-associated molecules through pattern-recognition receptors in a tightly controlled manner, thereby eliminating harmful stimuli while maintaining mucosal immune homeostasis (106). However, during the early stages of IBD, disruption of the intestinal mucosal barrier increases exposure to microbial products, rendering macrophages and dendritic cells more susceptible to activation (107). Studies have demonstrated that mucosal immune cells from patients with IBD can release markedly elevated levels of TNF-α and IL-1β even in response to low-dose lipopolysaccharide stimulation, suggesting a reduced threshold for immune activation (108).

At the molecular level, this process is closely associated with aberrant activation of pattern-recognition receptor–related signaling pathways. Inflammatory cascades centered on the NF-κB signaling pathway can be rapidly initiated, promoting the transcriptional expression of multiple pro-inflammatory genes (109). Concurrently, inflammatory signaling may participate in the modulation of DNA methylation status through the regulation of epigenetic modifiers (110). Previous studies have shown that inflammatory cytokines such as TNF-α can upregulate DNMT1 and DNMT3A expression, whereas oxidative stress conditions may influence TET family enzyme activity, thereby altering 5-hydroxymethylcytosine levels and contributing to DNA demethylation processes (111).

These epigenetic alterations may further affect the transcriptional regulation of immune-related genes. In the intestinal mucosa of patients with IBD, promoter regions of pattern-recognition receptor genes such as TLR4 have been reported to exhibit hypomethylation accompanied by increased gene expression, potentially enhancing microbial signal recognition and promoting inflammatory cytokine release (112). *In vitro* studies have further demonstrated that manipulation of DNA methylation levels can modulate the responsiveness of macrophages to inflammatory stimulation (113). Meanwhile, inflammation-driven hypomethylation may sustain persistent overexpression of related genes, maintaining immune cells in a hyperresponsive state and establishing an early immuno-epigenetic amplification loop during disease initiation (114).

#### 4.1.2 Mucosal barrier dysfunction

During the early stage of IBD development, disruption of intestinal mucosal barrier integrity is considered a major trigger of inflammatory responses (115). Under normal conditions, epithelial cells maintain barrier integrity through tight junction proteins and the mucus layer, thereby restricting the entry of luminal microbes and microbial products into the lamina propria (116). Studies have shown that the expression of tight junction proteins, including ZO-1 and Occludin, as well as mucus-associated molecules such as MUC2, is markedly reduced in patients with IBD, consistent with increased epithelial permeability and impaired barrier function (117).

Alterations in barrier architecture can substantially influence local immune activation patterns. As epithelial permeability increases, microbial-associated molecules gain easier access to mucosal tissues, leading to persistent activation of macrophages and dendritic cells and increased release of inflammatory cytokines such as TNF-α and IL-1β (118). Evidence further suggests that the severity of barrier disruption correlates with the magnitude of local inflammatory responses, supporting its critical role in inflammatory initiation (119).

Within this context, DNA methylation may contribute to the regulation of barrier-related gene expression. Previous studies have demonstrated that promoter regions of genes such as ZO-1, Occludin, and MUC2 exhibit increased methylation levels in intestinal mucosal tissues from patients with IBD, accompanied by transcriptional repression (120). In addition, inflammatory cytokines including TNF-α may further enhance methylation of these genes by regulating DNMT1 and DNMT3A expression, thereby sustaining their low-expression states (121). Simultaneously, persistent immune stimulation resulting from barrier disruption may further reshape epigenetic regulation. Oxidative stress within the inflammatory microenvironment has been proposed to influence TET enzyme activity, thereby participating in DNA demethylation processes and modulating related gene expression (122, 123).

#### 4.1.3 Early formation of epigenetic alterations

During the initiation phase of IBD, the emergence of an inflammatory microenvironment is accompanied by early remodeling of DNA methylation patterns, a process that exhibits cell type–specific characteristics (124). In intestinal macrophages and dendritic cells, inflammation-driven signaling pathways can influence the expression of epigenetic regulatory enzymes (125). For example, TNF-α and IL-6 may regulate DNMT1 and DNMT3A expression through NF-κB and JAK/STAT signaling pathways, thereby altering methylation states within promoter regions of selected inflammation-related genes and rendering them more transcriptionally permissive (126). Correspondingly, genes such as TLR4 and IL1B may remain highly expressed under hypomethylated conditions, enhancing the responsiveness of immune cells to microbial stimuli (127).

In epithelial cells, oxidative stress–associated pathways may participate in the regulation of DNA demethylation (128). Elevated levels of reactive oxygen species within the inflammatory microenvironment can influence TET family enzyme activity and alter 5-hydroxymethylcytosine abundance, thereby contributing to the regulation of barrier-related gene expression (129). Previous studies suggest that altered methylation states of MUC2 and tight junction–related genes are associated with reduced expression, weakened mucus layer integrity, and increased epithelial permeability (130). Moreover, in neutrophils and monocytes, expression changes in chemotaxis-related genes such as CXCL8 may also be linked to local methylation status, thereby promoting inflammatory cell recruitment and amplifying local inflammatory responses (131).

**Figure 4:**
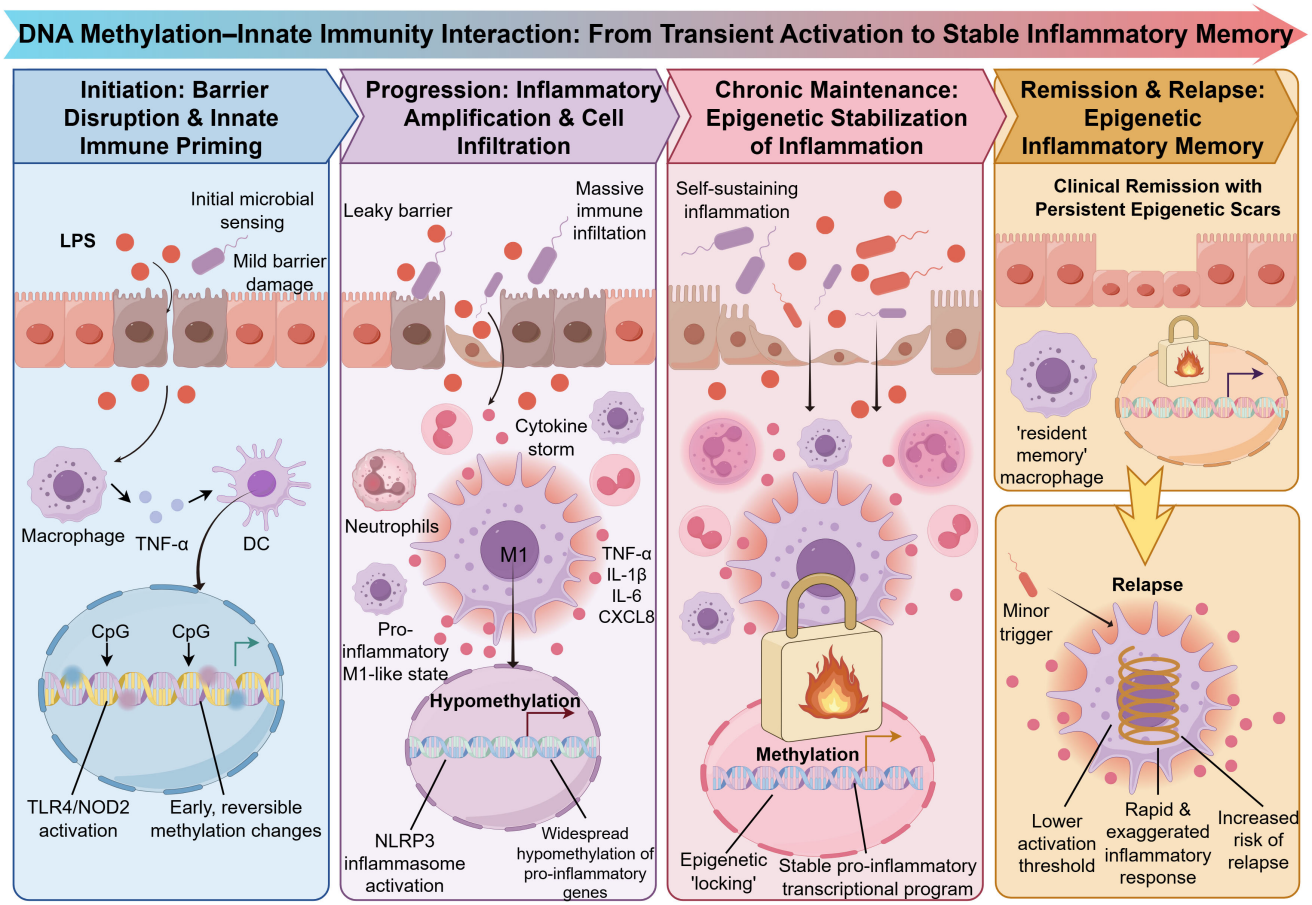
Dynamic roles of DNA methylation–innate immunity interactions during initiation, progression, chronic maintenance, and relapse of IBD. During disease initiation, barrier disruption and microbial stimulation trigger TLR4/NOD2 activation and early, potentially reversible methylation changes. During progression, immune cell infiltration, cytokine amplification, NLRP3 inflammasome activation, and widespread hypomethylation of pro-inflammatory genes amplify mucosal inflammation. As inflammation becomes chronic, DNA methylation changes may stabilize pro-inflammatory transcriptional programs and establish an epigenetic “locked” state. Even during clinical remission, some immune cells may retain memory-like inflammatory features, enabling rapid reactivation upon minor triggers and increasing relapse risk.

Overall, during the initiation phase of IBD, the DNA methylation–innate immunity interaction axis is primarily characterized by a reduced threshold for innate immune activation, aberrant methylation of barrier-related genes, and early epigenetic remodeling of inflammation-associated genes. At this stage, regulatory processes are still predominantly driven by microbial stimulation and local inflammatory signaling. Although the epigenetic alterations have not yet become fully stabilized, they progressively establish the foundation for reciprocal reinforcement between immune activation and epigenetic remodeling (**Figure 4**). Compared with physiological homeostasis, the defining feature of this stage is the emergence of persistent abnormalities in pattern-recognition receptor–related signaling pathways and barrier-associated regulatory mechanisms, thereby laying the groundwork for subsequent inflammatory expansion.

### 4.2 Disease progression stage

#### 4.2.1 Expansion of immune-cell infiltration

As IBD progresses from the initiation phase to the progression phase, inflammation gradually expands from localized immune activation to widespread immune cell recruitment and tissue infiltration. Studies have shown that circulating monocytes in patients with IBD continuously migrate to inflamed intestinal sites and differentiate into inflammatory macrophages, accompanied by a marked increase in neutrophils, resulting in a pro-inflammatory immune cell-dominant remodeling of mucosal immune composition (132). This reshaping of immune cell origin and proportion can drive the transition from local immune activation to a broader mucosal inflammatory response.

During this process, infiltrating immune cells sustain inflammatory signaling by releasing cytokines such as TNF-α, IL-1β, and IL-6 (133). Inflammatory regulatory networks centered on NF-κB and JAK/STAT signaling may remain activated across multiple immune cell populations. Meanwhile, activation of inflammasome-related molecules can further promote the maturation and release of inflammatory mediators, thereby amplifying the inflammatory response (134, 135).

DNA methylation also participates in immune cell infiltration and inflammatory expansion. Inflammation-related genes, including TNF, IL1B, and CXCL8, may exhibit hypomethylation in inflammatory cells, maintaining high transcriptional activity and enhancing inflammatory signal output (136). In addition, epigenetic regulation of chemokine-related genes may facilitate the recruitment of immune cells to inflamed sites, further expanding the inflammatory field (137). Cytokines and oxidative stress within the inflammatory microenvironment can also modulate DNMT and TET enzyme activity, thereby altering methylation states of relevant genes and sustaining the pro-inflammatory phenotype of infiltrating immune cells (138).

#### 4.2.2 Altered immune-cell function

As inflammation continues to progress, immune cell alterations are reflected not only by increased cell numbers but also by persistent functional reprogramming and phenotypic stabilization (139). At the macrophage level, the inflammatory microenvironment can drive polarization toward a pro-inflammatory phenotype. In the intestinal mucosa of patients with IBD, the proportion of inflammatory macrophages is increased, accompanied by upregulated inducible nitric oxide synthase (iNOS) expression and sustained release of inflammatory cytokines such as TNF-α and IL-1β. Conversely, the expression of anti-inflammatory molecules such as ARG1 and IL-10 is relatively reduced, suggesting impaired immunoregulatory capacity (140). This phenotypic shift is closely associated with persistent activation of NF-κB and JAK/STAT signaling pathways.

DNA methylation plays an important regulatory role in macrophage functional reprogramming. Pro-inflammatory genes, such as iNOS and TNF, may display hypomethylation in inflammatory macrophages and maintain elevated expression levels (141). In contrast, anti-inflammatory genes, such as ARG1 and IL10, may be transcriptionally suppressed due to increased methylation levels (142). This differential methylation pattern may help stabilize the pro-inflammatory phenotype and hinder the restoration of immune homeostasis.

Functional reprogramming is also evident in dendritic cells. IBD-associated dendritic cells tend to produce higher levels of pro-inflammatory cytokines such as IL-12, whereas secretion of immunoregulatory factors such as IL-10 is reduced, indicating impaired tolerogenic capacity (143, 144). Related studies suggest that these changes may be associated with altered methylation states of cytokine-related genes, thereby influencing the direction of local immune responses (145). In addition, neutrophils and monocytes also exhibit enhanced inflammatory functions. Sustained expression of chemokines and inflammatory mediators in these cells may be influenced by epigenetic regulation, further amplifying inflammatory responses (146, 147).

#### 4.2.3 Epigenetic stabilization and sustained activation of inflammatory responses

As disease further advances, inflammatory responses may gradually shift from being driven by external stimuli to a relatively stable state dominated by endogenous regulatory mechanisms. Studies have shown that in some patients with IBD, the expression of inflammation-related genes in the intestinal mucosa remains elevated even after external stimuli are reduced, suggesting that inflammatory responses may persist, at least in part, independently of the initial triggering factors (148).

In this process, DNA methylation may contribute to the stabilization of inflammation-associated gene expression states (149). Promoter regions of genes such as TNF, IL1B, and CXCL8 may exhibit sustained hypomethylation in inflamed tissues, maintaining a relatively open transcriptional configuration and persistently high expression levels (150). Unlike in the early disease stage, these epigenetic features more closely resemble a relatively stable transcriptional state rather than a transient response to inflammatory signals.

At the cellular level, such epigenetic stability may sustain persistent immune cell activation. Inflammatory macrophages and dendritic cells in the local microenvironment may display relatively stable pro-inflammatory transcriptional programs, with cytokine expression no longer fully dependent on exogenous stimulation (151). Meanwhile, the long-term presence of cytokines and oxidative stress within the inflammatory milieu may continuously regulate DNMT and TET enzyme activity, further consolidating these aberrant methylation states (152).

During the progression phase of IBD, inflammatory responses may gradually form stable expression patterns maintained by epigenetic regulation, keeping inflammation-related genes in a persistently activation-prone state and promoting self-sustaining inflammation (153). Compared with the initiation phase, the DNA methylation–innate immunity interaction axis at this stage has shifted from “initial activation” to “sustained amplification and stable maintenance.” Its core features include extensive inflammatory cell infiltration, stabilization of pro-inflammatory phenotypes, and the establishment of persistent hypomethylation in inflammation-associated genes such as TNF, IL1B, and CXCL8. At this point, NF-κB, JAK/STAT, and inflammasome-related signaling become coupled with DNMT/TET-mediated epigenetic remodeling, allowing inflammation to gradually become less dependent on transient exogenous stimuli and evolve into a more stable chronic inflammatory state. This DNA methylation-mediated persistent activation displays features of inflammatory memory to some extent and provides an important basis for the transition of inflammation from a reversible process to chronicity (154) (**Figure 4**).

### 4.3 Epigenetically driven inflammatory memory and relapse mechanisms

Inflammatory bowel disease is characterized by a chronic relapsing course. Even during clinical remission, intestinal mucosal gene expression profiles and immune regulatory features may not fully return to healthy baseline levels, suggesting that disease-associated regulatory alterations can persist beyond symptomatic recovery (155). Within this context, DNA methylation may contribute to the maintenance of inflammation-related transcriptional programs. Previous studies have shown that hypomethylation states established during disease progression in inflammation-associated genes such as TNF, IL1B, and CXCL8 may be partially retained during remission, thereby maintaining relatively high transcriptional activity at these loci (156). Compared with the progression phase of disease, these epigenetic alterations more closely resemble stable transcriptional regulatory states rather than transient responses to inflammatory signaling. Upon re-exposure to triggers such as microbial fluctuations or mild mucosal barrier disruption, these genes may therefore be rapidly reactivated, leading to re-initiation of inflammatory responses (157).

This regulatory feature is not limited to individual genes but may also manifest as broader alterations in immune system responsiveness. During remission, innate immune cells may retain partial pro-inflammatory transcriptional characteristics, including reduced activation thresholds and accelerated responses to external stimuli, thereby facilitating amplification of inflammatory signaling (158). Available evidence suggests that these alterations may result from epigenetic remodeling induced during prior inflammatory episodes, rather than relying solely on the persistent presence of exogenous stimuli (159).

In addition, the metabolic and stress-related microenvironment established during chronic inflammation may further contribute to the persistence of aberrant DNA methylation patterns. Long-term exposure to inflammatory cytokines and oxidative stress can continuously influence the activity of DNA methyltransferases and demethylation-associated enzymes, preventing certain methylation states from fully returning to baseline and thereby maintaining an increased inflammatory predisposition at the molecular level (160). Such a state may render the intestinal mucosa more sensitive to minor stimuli and increase susceptibility to disease relapse.

At the clinical level, several DNA methylation loci have been associated with relapse risk in patients with IBD, highlighting their potential value in disease activity assessment and prognostic evaluation (161). Collectively, these findings suggest that DNA methylation may sustain an aberrant transcriptional regulatory framework that preserves heightened immune responsiveness even during remission, thereby providing a molecular basis for recurrent inflammation.

During the remission and relapse phase, the DNA methylation–innate immunity interaction axis further acquires features of “inflammatory memory.” Compared with the progression stage, the intensity of local inflammation may decline; however, certain aberrant methylation patterns and pro-inflammatory transcriptional programs persist, maintaining heightened sensitivity of the innate immune system. Upon renewed exposure to microbial fluctuations, mild barrier injury, or localized inflammatory stimuli, inflammation-associated pathways can be rapidly reactivated, thereby promoting disease recurrence (**Figure 4**). This stage further suggests that epigenetic abnormalities in IBD are not only involved in the initiation and maintenance of inflammation, but may also constitute an important molecular foundation underlying chronic relapse.

## 5 Therapeutic strategies based on DNA methylation–innate immunity crosstalk

### 5.1 Integration and mechanistic features of current therapeutic strategies

From the perspective of DNA methylation–innate immunity crosstalk, current therapeutic strategies for IBD can be integrated across multiple regulatory levels (**Table 2**). Overall, existing interventions mainly focus on suppressing inflammatory signaling and modulating immune function, whereas therapies that directly target DNA methylation remain relatively limited. Notably, several immunomodulatory agents have been shown to participate in disease regulation by influencing epigenetic regulatory networks while exerting anti-inflammatory effects.

**Table 2:** Potential therapeutic strategies and mechanistic features based on the DNA methylation–innate immunity crosstalk axis in IBD.

Regulatory level	Intervention/agent	Mechanism of action	Effect on innate immunity	Clinical progress	References
Epigenetic regulation	5-Azacytidine, Decitabine	Inhibit DNA methyltransferase activity, reduce DNA methylation levels, and restore the expression of related genes	Upregulate anti-inflammatory genes and attenuate inflammatory responses	Clinically used in oncology; preclinical stage in IBD	(162, 163)
Enhancement of epigenetic regulation	Vitamin C	Promotes TET enzyme-mediated DNA demethylation	Regulates the balance of inflammation-related gene expression	Experimental studies	(164)
Inflammatory signaling regulation	NF-κB inhibitors	Inhibit activation of the NF-κB signaling pathway and influence epigenetic regulatory networks	Reduce the release of TNF-α and IL-1β	Preclinical/partial clinical evidence	(165)
Inflammatory signaling regulation	Tofacitinib	Inhibits the JAK/STAT signaling pathway and regulates the expression of inflammation-related genes	Suppresses excessive activation of immune cells	Approved/used for IBD treatment	(166)
Pathogen-recognition regulation	TLR4 inhibitors	Inhibit TLR4-mediated inflammatory signaling and affect downstream gene-expression regulation	Reduce the threshold for immune activation	Animal studies	(167)
Inflammasome regulation	NLRP3 inhibitors	Inhibit inflammasome activation and regulate inflammatory gene expression	Reduce IL-1β release	Experimental studies	(168, 169)
Immune-function modulation	Immunomodulators	Regulate the expression and epigenetic status of macrophage polarization-related genes	Promote restoration of immune balance	Experimental studies	(170, 171)

From an overall mechanistic perspective, although different therapeutic strategies target distinct pathways, they exhibit convergent effects in the regulation of inflammatory signaling and immune cell function. Agents targeting inflammatory signaling pathways often suppress cytokine release while simultaneously altering the transcriptional states of inflammation-associated genes, and these changes may be closely linked to remodeling of the epigenetic regulatory environment (172). Reduced inflammatory signaling intensity can influence the expression and activity of DNA methylation–related enzymes, thereby modulating the transcription of inflammation-related genes at the epigenetic level.

Regulation of immune cell function may not only alter the immediate activation state of immune cells, but also affect the stability of related gene expression programs, enabling cellular phenotypes to maintain relatively consistent functional characteristics over time (171). This transition from functional modulation to sustained transcriptional regulation further suggests a close interplay between immune regulation and epigenetic states.

In addition, interactions among distinct regulatory layers contribute to the network-like nature of therapeutic effects. Interventions targeting a single pathway may simultaneously exert indirect effects on other regulatory components, thereby generating cumulative and interconnected effects across the broader regulatory network. This multilayered mode of interaction may help explain the broad biological impact observed with certain therapeutic strategies, while also highlighting the potential limitations of single-target interventions within such a complex regulatory system (172).

### 5.2 Limitations

Although accumulating evidence has revealed interactions between DNA methylation and innate immunity in IBD at multiple levels, several important limitations remain. First, current evidence is still relatively insufficient in terms of population-based studies, longitudinal follow-up investigations, and functional validation, and discrepancies persist among studies regarding specific methylation loci and regulatory directions. Second, DNA methylation exhibits pronounced cell type specificity, yet most existing studies remain unable to precisely dissect differential regulatory patterns across distinct cellular populations. This cellular heterogeneity is particularly evident in intestinal macrophages. Resident intestinal macrophages and monocyte-derived recruited inflammatory macrophages differ substantially in cellular origin, turnover dynamics, inflammatory function, and adaptation to the local microenvironment, and their DNA methylation profiles and inflammation-related transcriptional programs may therefore also differ considerably.

In addition, the temporal sequence and causal relationship between DNA methylation alterations and innate immune activation remain to be further clarified. Aberrant methylation at certain inflammation- or barrier-associated CpG loci has been linked to IBD disease activity, inflammatory severity, and relapse risk. However, most current evidence is derived from cross-sectional tissue analyses, *in vitro* stimulation experiments, or acute animal models, which are insufficient to fully capture the continuous pathogenic process spanning early epigenetic alterations, immune activation, and subsequent tissue inflammatory injury.

Finally, current mechanistic evidence remains limited in terms of model translatability and therapeutic applicability. Although DSS-induced acute colitis models and LPS-stimulated *in vitro* cellular systems are useful for investigating fundamental mechanisms, they cannot fully recapitulate the long-term chronicity, recurrent relapses, and remission–flare cycles characteristic of human IBD. DNMT/TET-targeted interventions still face several translational bottlenecks, including insufficient locus and cell-type specificity, limited delivery efficiency, potential genome-wide off-target effects, and uncertain long-term safety. How to correct aberrant inflammatory epigenetic programs without disrupting normal immune homeostasis remains a key challenge for future clinical translation.

### 5.3 Emerging precision epigenetic–immune intervention strategies and future directions

Elucidating the regulatory interplay between DNA methylation and innate immunity, and translating these insights into clinical applications, represents an important direction for future research. On the one hand, future studies should integrate technologies such as single-cell sequencing, spatial transcriptomics, and single-cell methylomics to dissect cell type–specific DNA methylation regulatory patterns in immune and epithelial cells. Particular attention should be given to clarifying the epigenetic heterogeneity and dynamic interactions among distinct innate immune cell subsets, including resident macrophages and recruited inflammatory macrophages. In parallel, longitudinal cohort studies and time-course animal experiments are needed to dynamically evaluate the temporal sequence and potential causal relationships between DNA methylation alterations and inflammatory responses.

From the perspective of therapeutic translation, future strategies should gradually shift from conventional broad-spectrum immunosuppression toward more precise epigenetic–immune co-regulation. With the development of epigenetic editing technologies, locus-specific methylation editing based on CRISPR/dCas9 systems may emerge as a promising approach. By fusing dCas9 with DNMT- or TET-associated functional domains, it may theoretically become possible to selectively induce methylation or demethylation at promoter regions of inflammation-related genes such as TLR4, TNF, IL1B, and CXCL8, thereby precisely modulating inflammatory response thresholds and cytokine release in innate immune cells. Compared with conventional broad-spectrum DNMT inhibitors, this locus-specific strategy may reduce nonspecific genome-wide effects and improve both safety and targeting specificity.

Beyond locus-directed editing, the development of small-molecule strategies targeting specific DNMT or TET isoforms may also hold considerable therapeutic potential. Currently available agents such as 5-Azacytidine and Decitabine primarily exert their effects through global inhibition of DNA methylation, and long-term administration may be associated with off-target effects, disruption of immune homeostasis, and safety concerns (162–164). In contrast, selective modulation of DNMT1, DNMT3A, or specific TET isoforms may allow more precise intervention against inflammation-associated epigenetic abnormalities in IBD while minimizing adverse effects on normal cellular function.

In addition, patient stratification based on DNA methylation characteristics may become an important component of future precision medicine strategies. Significant heterogeneity likely exists among patients with IBD regarding methylation patterns of inflammation-related genes, barrier-associated genes, and immune regulatory loci, and these differences may influence responses to anti-TNF biologics, JAK inhibitors, and other immunotherapies. Future integration of DNA methylation profiles, immune cell states, and clinical phenotypes into predictive models may help optimize therapeutic selection, improve biologic response rates, and identify individuals at high risk of relapse.

It should be noted that most of these approaches remain at the experimental or early exploratory stage, and their delivery efficiency, cell specificity, long-term safety, and clinical feasibility require further validation. Nevertheless, these emerging strategies suggest that future IBD therapy may gradually evolve from simply suppressing inflammatory signaling toward precise coordinated regulation targeting the “DNA methylation–innate immunity interaction axis,” thereby providing novel therapeutic avenues for chronic inflammatory maintenance and disease recurrence.

## 6 Conclusion

The pathogenesis and progression of inflammatory bowel disease involve a multilayered regulatory network, within which the interaction between DNA methylation and the innate immune system constitutes a critical mechanistic foundation. Current evidence indicates that DNA methylation not only participates in the transcriptional regulation of immune-related genes, but also influences pathogen recognition, inflammatory signal transduction, immune cell functional modulation, and maintenance of the mucosal barrier. At the same time, innate immune responses can reciprocally reshape DNA methylation states through inflammatory cytokines, oxidative stress, and associated signaling pathways, thereby establishing a dynamic regulatory interplay throughout disease progression.

From the perspective of disease evolution, the DNA methylation–innate immunity interaction exhibits stage-specific characteristics during inflammatory initiation, progression, and relapse, and may contribute to the sustained amplification and stabilization of inflammatory responses. Accordingly, re-integrating current therapeutic strategies through the framework of the DNA methylation–innate immunity interaction axis may deepen mechanistic understanding and provide a theoretical basis for optimizing future intervention strategies.

Although important limitations remain in current research, ongoing advances in multi-omics technologies and methodological approaches are expected to further clarify the complex interplay between epigenetic regulation and immune responses. In this context, combined interventions targeting both epigenetic remodeling and immune dysregulation may offer promising new directions for precision therapy in IBD.

## References

[1] Hu Y Yang Y Li Y Zhang Q Zhang W Jia J . Th17/Treg imbalance in inflammatory bowel disease: immunological mechanisms and microbiota-driven regulation. Front Immunol. (2025) 16:1651063. doi: 10.3389/fimmu.2025.1651063 41132656 PMC12540096

[2] Kumar M Murugesan S Ibrahim N Elawad M Al Khodor S . Predictive biomarkers for anti-TNF alpha therapy in IBD patients. J Transl Med. (2024) 22:284. doi: 10.1186/s12967-024-05058-1 38493113 PMC10943853

[3] Jairath V Narula N Ungaro RC Romo Bautista I Adsul S . Novel outcomes in inflammatory bowel disease. J Crohns Colitis. (2025) 19:jjaf040. doi: 10.1093/ecco-jcc/jjaf040 40078047 PMC12032607

[4] Grondin JA Kwon YH Far PM Haq S Khan WI . Mucins in intestinal mucosal defense and inflammation: learning from clinical and experimental studies. Front Immunol. (2020) 11:2054. doi: 10.3389/fimmu.2020.02054 33013869 PMC7500085

[5] Zhou X Wu Y Zhu Z Lu C Zhang C Zeng L . Mucosal immune response in biology, disease prevention and treatment. Signal Transd Targ Ther. (2025) 10:7. doi: 10.1038/s41392-024-02043-4 39774607 PMC11707400

[6] Wang J He M Yang M Ai X . Gut microbiota as a key regulator of intestinal mucosal immunity. Life Sci. (2024) 345:122612. doi: 10.1016/j.lfs.2024.122612 38588949

[7] Meng W Fenton CG Johnsen KM Taman H Florholmen J Paulssen RH . DNA methylation fine-tunes pro-and anti-inflammatory signalling pathways in inactive ulcerative colitis tissue biopsies. Sci Rep. (2024) 14:6789. doi: 10.1038/s41598-024-57440-0 38514698 PMC10957912

[8] Guerin LN Scott TJ Yap JA Johansson A Puddu F Charlesworth T . Temporally discordant chromatin accessibility and DNA demethylation define short- and long-term enhancer regulation during cell fate specification. Cell Rep. (2025) 44:115680. doi: 10.1016/j.celrep.2025.115680 40349339 PMC12153380

[9] Johnston RA Aracena KA Barreiro LB Lea AJ Tung J . DNA methylation-environment interactions in the human genome. Elife. (2024) 12:RP89371. doi: 10.7554/eLife.89371 38407202 PMC10942648

[10] Chen J Qi D Hu H Wang X Lin W . Unconventional posttranslational modification in innate immunity. Cell Mol Life Sci. (2024) 81:290. doi: 10.1007/s00018-024-05319-8 38970666 PMC11335215

[11] Liu Z Kilic G Li W Bulut O Gupta MK Zhang B . Multi-Omics integration reveals only minor long-term molecular and functional sequelae in immune cells of individuals recovered from COVID-19. Front Immunol. (2022) 13:838132. doi: 10.3389/fimmu.2022.838132 35464396 PMC9022455

[12] Chen PR Li CY Yazal T Chen IC Liu PL Chen YT . Protective effects of nordalbergin against LPS-induced endotoxemia through inhibiting MAPK/NF-κB signaling pathway, NLRP3 inflammasome activation, and ROS production. Inflammation Res. (2024) 73:1657–70. doi: 10.1007/s00011-024-01922-4 39052062

[13] Guo X Li J Xu J Zhang L Huang C Nie Y . Gut microbiota and epigenetic inheritance: implications for the development of IBD. Gut Microbes. (2025) 17:2490207. doi: 10.1080/19490976.2025.2490207 40213833 PMC12931708

[14] Rioux JD Boucher G Forest A Bouchard B Coderre L Daneault C . Serum proteomic and metabolomic analyses from patients with IBD identify biological pathways associated with treatment success with anti-integrin therapy. Immunol Cell Biol. (2025) 103:648–63. doi: 10.1111/imcb.70039 40509637 PMC12354290

[15] Tse AY Spakowitz AJ . Modeling DNA methyltransferase function to predict epigenetic correlation patterns in healthy and cancer cells. Proc Natl Acad Sci USA. (2025) 122:e2415530121. doi: 10.1073/pnas.2415530121 39792289 PMC11745332

[16] Chai X Wang H Wang B Ma Y Zhang X Guo J . Mendelian randomization integrated with multi-omics analysis identifies TNIK as a key gene in gut microbiota-induced IBD development. Front Immunol. (2025) 16:1678444. doi: 10.3389/fimmu.2025.1678444 41341599 PMC12669205

[17] Shilkin ES Petrova DV Novikova AA Boldinova EO Zharkov DO Makarova AV . Methylation and hydroxymethylation of cytosine alter activity and fidelity of translesion DNA polymerases. DNA Repair (Amst). (2024) 141:103712. doi: 10.1016/j.dnarep.2024.103712 38959714

[18] Zacapala-Gómez AE Mendoza-Catalán MA Antonio-Véjar V Jiménez-Wences H Ortíz-Ortíz J Ávila-López PA . TET enzymes and 5hmC epigenetic mark: new key players in carcinogenesis and progression in gynecological cancers. Eur Rev Med Pharmacol Sci. (2024) 28:1123–34. doi: 10.26355/eurrev_202402_35349 38375718

[19] Neikes HK Kliza KW Gräwe C Wester RA Jansen PWTC Lamers LA . Quantification of absolute transcription factor binding affinities in the native chromatin context using BANC-seq. Nat Biotechnol. (2023) 41:1801–9. doi: 10.1038/s41587-023-01715-w 36973556

[20] Xie S Hagen D Becker GM Davenport KM Shira KA Stegemiller MR . Analyzing the relationship of RNA and DNA methylation with gene expression. Genome Biol. (2025) 26:140. doi: 10.1186/s13059-025-03617-3 40405312 PMC12101012

[21] Wang Y Zhang J Li W Jiang Y Xu X Jiang Q . DNA methylation signature of oxidative stress and its mediating role in response to metal exposure. Free Radic Biol Med. (2025) 240:211–21. doi: 10.1016/j.freeradbiomed.2025.08.039 40840620

[22] Doherty T McDermott E Delany SJ Mulcahy H Murphy TM . Analysis of blood-based DNA methylation signatures of aging and disease progression in inflammatory bowel disease. Hum Genet. (2025) 144:1079–95. doi: 10.1007/s00439-025-02779-1 41020989 PMC12689681

[23] Mahurkar-Joshi S Thompson M Villarruel E Lewis JD Lin LD Farid M . Genome-wide DNA methylation identifies potential disease-specific biomarkers and pathophysiologic mechanisms in irritable bowel syndrome, inflammatory bowel disease, and celiac disease. Neurogastroenterol Motil. (2025) 37:e14980. doi: 10.1111/nmo.14980 39673136 PMC11748828

[24] Gribkova AK Bigildeev AE Shaytan AK . The methylation level of a CpG site in the human interleukin-1β promoter reflects both current and past inflammation. Russ J Genet. (2024) 60:962–8. doi: 10.1134/S1022795424700406

[25] Meng M Ma Y Xu J Chen G Mahato RK . DNA methylation-mediated FGFR1 silencing enhances NF-κB signaling: implications for asthma pathogenesis. Front Mol Biosci. (2024) 11:1433557. doi: 10.3389/fmolb.2024.1433557 39377013 PMC11456769

[26] Zhao J Yao W Gao H Kuang Z Shi L Wang H . Degenerative disease diagnosis and analysis based on tissue specificity of DNA methylation. Int J Mol Sci. (2025) 26:452. doi: 10.3390/ijms26020452 39859168 PMC11765164

[27] Joustra VW Li Yim AYF Henneman P Hageman I de Waard T Levin E . Development and validation of peripheral blood DNA methylation signatures to predict response to biological therapy in adults with Crohn's disease (EPIC-CD): an epigenome-wide association study. Lancet Gastroenterol Hepatol. (2025) 10:818–30. doi: 10.1016/S2468-1253(25)00102-5 40614748

[28] Zhang H Kalla R Chen J Zhao J Zhou X Adams A . Altered DNA methylation within DNMT3A, AHRR, LTA/TNF loci mediates the effect of smoking on inflammatory bowel disease. Nat Commun. (2024) 15:595. doi: 10.1038/s41467-024-44841-y 38238335 PMC10796384

[29] Meier HCS Klopack ET Farnia MP Hernandez B Mitchell C Faul JD . A novel DNA methylation-based surrogate biomarker for chronic systemic inflammation (Inflammation Latent Variable Methylation Surrogate, InfLaMeS). J Gerontol A Biol Sci Med Sci. (2025) 80:glaf202. doi: 10.1093/gerona/glaf202 40990779 PMC13047507

[30] Pashos ARS Meyer AR Bussey-Sutton C O'Connor ES Coradin M Coulombe M . H3K36 methylation regulates cell plasticity and regeneration in the intestinal epithelium. Nat Cell Biol. (2025) 27:202–17. doi: 10.1038/s41556-024-01580-y 39779942 PMC12342706

[31] Hiebert P Werner S . Targeting NRF2 to promote epithelial repair. Biochem Soc Trans. (2023) 51:101–11. doi: 10.1042/BST20220228 36762597 PMC9987932

[32] Johnson AA Shokhirev MN . Demystifying common DNA methylation sites that promote the ability of CheekAge to associate with health and disease. Ageing Res Rev. (2025) 111:102839. doi: 10.1016/j.arr.2025.102839 40691975

[33] Ma Y Wang X Li X . The emerging role of DNA methylation in the pathogenicity of bacterial pathogens. J Bacteriol. (2025) 207:e0010825. doi: 10.1128/jb.00108-25 40673666 PMC12369329

[34] Jhuang KF Hsu ML Chen YC Chang JG Zouali M . DNA methylation trajectories during innate and adaptive immune responses of human B lymphocytes. Immunology. (2023) 169:344–57. doi: 10.1111/imm.13632 36762485

[35] Sinke L van Dongen J Delerue T Wilson R Xia Y Beekman M . Epigenome-wide association study of circulating interleukin-6 connects DNA methylation to immunometabolic and inflammatory health. Commun Biol. (2026) 9:242. doi: 10.1038/s42003-026-09520-2 41639309 PMC12905258

[36] Li M Zhang Y Jiang W Li S Lin X Ma M . ID1 boosts antiviral immunity by countering PRMT5-mediated STING methylation. Cell Rep. (2025) 44:116547. doi: 10.1016/j.celrep.2025.116547 41205173

[37] McBride MA Caja KR Patil TK Owen AM Luan L Bohannon JK . Immunoresponsive gene 1 facilitates TLR4 agonist-induced augmentation of innate antimicrobial immunity. J Leukoc Biol. (2025) 117:qiae198. doi: 10.1093/jleuko/qiae198 39351765 PMC11879002

[38] Zúquete S Ferreira M Delgado ILS Gazalle P Andaluz S Rosa MT . Combined TLR2/TLR4 activation equip non-mucosal dendritic cells to prime Th1 cells with gut tropism. iScience. (2024) 27:111232. doi: 10.1016/j.isci.2024.111232 39759015 PMC11700634

[39] Anacona CA Álvarez K Vásquez G Rojas M . Regulatory effects of CD14+CD16++Slan+ monocytes on classical monocytes and T lymphocytes in response to inflammatory and phagocytic stimuli. J Immunol Res. (2026) 2026:e3322498. doi: 10.1155/jimr/3322498 41964644 PMC13581061

[40] Zhu X Zhang L Feng D Jiang L Sun P Zhao C . A LY6E-PHB1-TRIM21 assembly degrades CD14 protein to mitigate LPS-induced inflammatory response. iScience. (2023) 26:106808. doi: 10.1016/j.isci.2023.106808 37250795 PMC10209397

[41] Li X Zhao C . Interleukin-6 in neuroimmunological disorders: pathophysiology and therapeutic advances with satralizumab. Autoimmun Rev. (2025) 24:103826. doi: 10.1016/j.autrev.2025.103826 40324548

[42] Lou X Duan S Li M Yuan Y Chen S Wang Z . IL-36α inhibits melanoma by inducing pro-inflammatory polarization of macrophages. Cancer Immunol Immunother. (2023) 72:3045–61. doi: 10.1007/s00262-023-03477-5 37318520 PMC10992341

[43] Huisman JMA Drakaki A Decker T Hoeksema MA . Cytokine specificity in macrophages: JAK-STAT and beyond. Trends Immunol. (2026) 47:255–8. doi: 10.1016/j.it.2026.01.002 41763992

[44] Sawoo R Bishayi B . TLR4/TNFR1 blockade suppresses STAT1/STAT3 expression and increases SOCS3 expression in modulation of LPS-induced macrophage responses. Immunobiology. (2024) 229:152840. doi: 10.1016/j.imbio.2024.152840 39126792

[45] Yu H Sun X Li Y Pan J Liu X He H . Macrophage NLRP3 activation and IL-1β release drive osimertinib-induced antitumor immunity. J Immunother Cancer. (2025) 13:e012182. doi: 10.1136/jitc-2025-012182 41052880 PMC12506461

[46] Perez DC Hernandez-Franco JF HogenEsch H . Aluminum adjuvants differentially induce IL-1β release *in vitro* yet share NLRP3 inflammasome-independent adjuvant effects *in vivo*. Sci Rep. (2026) 16:4570. doi: 10.1038/s41598-025-34660-6 41519970 PMC12868721

[47] Gan YQ Cai YW Liang XW Wang LZ Shi FL Sun N . Impaired NLRP3 inflammasome signaling diverts pyroptotic to apoptotic caspase activation in macrophages. Front Immunol. (2025) 16:1631152. doi: 10.3389/fimmu.2025.1631152 41208996 PMC12591955

[48] Sharma BR Choudhury SM Abdelaal HM Wang Y Kanneganti TD . Innate immune sensor NLRP3 drives PANoptosome formation and PANoptosis. J Immunol. (2025) 214:1236–46. doi: 10.1093/jimmun/vkaf042 40249072 PMC12207079

[49] Kang JH Kim JH Gim JA Lee MY . iNOS in macrophage polarization: pharmacological and regulatory insights. Int J Mol Sci. (2025) 26:12056. doi: 10.3390/ijms262412056 41465487 PMC12733050

[50] John SV Seim GL Erazo-Flores BJ Votava JA Urquiza US Arp NL . Classically activated macrophages undergo functionally significant nucleotide metabolism remodelling driven by nitric oxide. Nat Metab. (2025) 7:1681–702. doi: 10.1038/s42255-025-01337-3 40759751 PMC12356500

[51] Min KY Kim DK Jo MG Choi MY Lee D Park JW . IL-27-induced PD-L1highSca-1+ innate lymphoid cells suppress contact hypersensitivity in an IL-10-dependent manner. Exp Mol Med. (2024) 56:616–29. doi: 10.1038/s12276-024-01187-1 38424193 PMC10984996

[52] Gilmour BC Corthay A Øynebråten I . High production of IL-12 by human dendritic cells stimulated with combinations of pattern-recognition receptor agonists. NPJ Vaccines. (2024) 9:83. doi: 10.1038/s41541-024-00869-1 38702320 PMC11068792

[53] Aboseif A Mangioris G Yang B Pazdernik VK Britton JW Dubey D . Cytokine, chemokine, and neurofilament light chain signatures in LGI1 autoimmune encephalitis. Ann Clin Transl Neurol. (2025) 12:2258–70. doi: 10.1002/acn3.70158 40781580 PMC12623847

[54] Madej M Halama A Chrobak E Gola JM . Time-dependent impact of betulin and its derivatives on IL-8 expression in colorectal cancer cells with molecular docking studies. Int J Mol Sci. (2025) 26:6186. doi: 10.3390/ijms26136186 40649964 PMC12250247

[55] Yu S He J Xie K . Zonula occludens proteins signaling in inflammation and tumorigenesis. Int J Biol Sci. (2023) 19:3804–15. doi: 10.7150/ijbs.85765 37564207 PMC10411466

[56] Raya-Sandino A Lozada-Soto KM Rajagopal N Garcia-Hernandez V Luissint AC Brazil JC . Claudin-23 reshapes epithelial tight junction architecture to regulate barrier function. Nat Commun. (2023) 14:6214. doi: 10.1038/s41467-023-41999-9 37798277 PMC10556055

[57] Gorman H Moreau F Beaupré E Nitin N Zandberg WF Bergstrom K . Using MUC2 mucin producing tumorigenic human goblet-like cells to uncover functional properties of the mucus barrier. Gut Microbes. (2025) 17:2542385. doi: 10.1080/19490976.2025.2542385 40781862 PMC12931731

[58] Gorman H Moreau F Dufour A Chadee K . IgGFc-binding protein and MUC2 mucin produced by colonic goblet-like cells spatially interact non-covalently and regulate wound healing. Front Immunol. (2023) 14:1211336. doi: 10.3389/fimmu.2023.1211336 37359538 PMC10285406

[59] Akhter N Wilson A Arefanian H Thomas R Kochumon S Al-Rashed F . Endoplasmic reticulum stress promotes the expression of TNF-α in THP-1 cells by mechanisms involving ROS/CHOP/HIF-1α and MAPK/NF-κB pathways. Int J Mol Sci. (2023) 24:15186. doi: 10.3390/ijms242015186 37894865 PMC10606873

[60] Tam FF Ning KL Lee M Dumlao JM Choy JC . Cytokine induction of HIF-1α during normoxia in A549 human lung carcinoma cells is regulated by STAT1 and JNK signalling pathways. Mol Immunol. (2023) 160:12–9. doi: 10.1016/j.molimm.2023.06.001 37295053

[61] Balaramane D Spill YG Weber M Bardet AF . MethyLasso: a segmentation approach to analyze DNA methylation patterns and identify differentially methylated regions from whole-genome datasets. Nucleic Acids Res. (2024) 52:e98. doi: 10.1093/nar/gkae880 39420630 PMC11602171

[62] Feng Z Cheng Y Wang Y Qu S Du J Gao F . Roxadustat protect mice from DSS-induced colitis *in vivo* by up-regulation of TLR4. Genomics. (2023) 115:110585. doi: 10.1016/j.ygeno.2023.110585 36801437

[63] Park J Luo Y Park JW Kim SH Hong YJ Lim Y . Downregulation of DNA methylation enhances differentiation of THP-1 cells and induces M1 polarization of differentiated macrophages. Sci Rep. (2023) 13:13132. doi: 10.1038/s41598-023-40362-8 37573395 PMC10423279

[64] Song M Li J Sun J Yang X Zhang X Lv K . DNMT1-mediated DNA methylation in toll-like receptor 4 (TLR4) inactivates NF-κB signal pathway-triggered pyroptotic cell death and cellular inflammation to ameliorate lipopolysaccharides (LPS)-induced osteomyelitis. Mol Cell Probes. (2023) 71:101922. doi: 10.1016/j.mcp.2023.101922 37459905

[65] Liang G He J Chen T Zhang L Yu K Shen W . Identification of ALDH7A1 as a DNA-methylation-driven gene in lung squamous cell carcinoma. Ann Med. (2025) 57:2442529. doi: 10.1080/07853890.2024.2442529 39711312 PMC11703541

[66] Yu W Li R Wang Y Wang YN Huang Y Zhang Y . MBD2 deficiency attenuates CCl4-induced hepatic fibrosis by inhibiting M2 macrophage polarization. Int Immunopharmacol. (2025) 164:115306. doi: 10.1016/j.intimp.2025.115306 40773897

[67] Mahdi FS Lillyman DJ Ney KE Wachs RA . Novel method to assess macrophage phenotype using eluted media. Cells Tissue Organs. (2025) 214:329–40. doi: 10.1159/000543141 39701063

[68] Akagha MJ Georgolopoulos G Martin D Meissl K Amenitsch L Vogl C . IFNγ shapes macrophage inflammatory responses by STAT1 isoform-specific epigenetic and transcriptional mechanisms. BMC Genomics. (2026) 27:256. doi: 10.1186/s12864-026-12601-5 41645061 PMC12973885

[69] Asgari F Nikzamir A Baghaei K Salami S Masotti A Rostami-Nejad M . Immunomodulatory and anti-inflammatory effects of vitamin A and tryptophan on monocyte-derived dendritic cells stimulated with gliadin in celiac disease patients. Inflammation. (2024) 47:1706–27. doi: 10.1007/s10753-024-02004-7 38492186

[70] Ghosh S Bishayi B . Neutralization of IL-17 and CXCR1 protects septic arthritis by regulating CXCL8-CXCR1 pathway along with functional activities in neutrophils. Int J Rheum Dis. (2025) 28:e70144. doi: 10.1111/1756-185X.70144 40195600

[71] Cao R Zhou J Liu J Wang Y Dai Y Jiang Y . TXM-CB13 improves the intestinal mucosal barrier and alleviates colitis by inhibiting the ROS/TXNIP/TRX/NLRP3 and TLR4/MyD88/NF-κB/NLRP3 pathways. Inflammation. (2025) 48:3529–41. doi: 10.1007/s10753-025-02282-9 40085192 PMC12596331

[72] Yi YS . Functional interplay between methyltransferases and inflammasomes in inflammatory responses and diseases. Int J Mol Sci. (2021) 22:7580. doi: 10.3390/ijms22147580 34299198 PMC8306412

[73] Han X Allaire JM Crowley SM Chan JJ Lau K Zhang C . Inflammasome activation links enteric Salmonella Typhimurium infection to a rapid, cytokine-dependent increase in intestinal mucin release. Gut Microbes. (2024) 16:2413372. doi: 10.1080/19490976.2024.2413372 39428744 PMC11497969

[74] Zhang T Lin Q Xu WY He Q Gong C Wang L . Mucosal DNA methylation reveals immune-related methylation profile and correlates with Crohn's disease status. Sci Rep. (2026) 16:2848. doi: 10.1038/s41598-025-29123-x 41571701 PMC12827257

[75] Zhou Q Li X Ma X Gao Y Li Y Xie H . Structural characterization of a neutral heteropolysaccharide from Pholidota chinensis Lindl. and its protective effects against intestinal inflammation via barrier enhancement and gut microbiota modulation. Int J Biol Macromol. (2025) 325:147181. doi: 10.1016/j.ijbiomac.2025.147181 40889656

[76] Meng EX Verne GN Zhou Q . Macrophages and gut barrier function: Guardians of gastrointestinal health in post-inflammatory and post-infection responses. Int J Mol Sci. (2024) 25:9422. doi: 10.3390/ijms25179422 39273369 PMC11395020

[77] Gao T Zhang H Xu Y He G Ma H Zheng C . HIF-1α enhances intestinal injury and inflammation in severe acute pancreatitis through NLRP3 inflammasome activation. Dig Dis Sci. (2025) 70:1813–23. doi: 10.1007/s10620-025-08926-y 39998719

[78] Yue Y Ren Y Lu C Li P Zhang G . Epigenetic regulation of human FOXP3+ Tregs: from homeostasis maintenance to pathogen defense. Front Immunol. (2024) 15:1444533. doi: 10.3389/fimmu.2024.1444533 39144146 PMC11323565

[79] Yang S Li X Bao L Cui J Liu J Cong S . Evolutionary and structural insights into DNMTs and TETs: decoding their functional heterogeneity and oncogenic roles in methylation regulation. BMC Mol Cell Biol. (2025) 26:26. doi: 10.1186/s12860-025-00552-w 40877782 PMC12392533

[80] Alfardan AS Nadeem A Ahmad SF Al-Harbi NO Alqinyah M Attia SM . DNMT inhibitor, 5-aza-2'-deoxycytidine mitigates di(2-ethylhexyl) phthalate-induced aggravation of psoriasiform inflammation in mice via reduction in global DNA methylation in dermal and peripheral compartments. Int Immunopharmacol. (2024) 137:112503. doi: 10.1016/j.intimp.2024.112503 38906008

[81] Salem S Mosaad R Lotfy R Elbadry M . Altered expression of DNA methyltransferases and methylation status of the TLR4 and TNF-α promoters in COVID-19. Arch Virol. (2023) 168:95. doi: 10.1007/s00705-023-05722-9 36840831 PMC9959945

[82] Ding J Jiang H Su B Wang S Chen X Tan Y . DNMT1/miR-130a/ZEB1 regulatory pathway affects the inflammatory response in lipopolysaccharide-induced sepsis. DNA Cell Biol. (2022) 41:479–86. doi: 10.1089/dna.2021.1060 35486848

[83] Zhu D Zeng S Su C Li J Xuan Y Lin Y . The interaction between DNA methylation and tumor immune microenvironment: from the laboratory to clinical applications. Clin Epigenet. (2024) 16:24. doi: 10.1186/s13148-024-01633-x 38331927 PMC10854038

[84] García-Giménez JL Cánovas-Cervera I Nacher-Sendra E Dolz-Andrés E Sánchez-Bernabéu Á Agúndez AB . Oxidative stress and central metabolism pathways impact epigenetic modulation in inflammation and immune response. Free Radic Biol Med. (2025) 233:378–99. doi: 10.1016/j.freeradbiomed.2025.04.004 40185167

[85] Chen Y Shen YQ . Role of reactive oxygen species in regulating epigenetic modifications. Cell Signal. (2025) 125:111502. doi: 10.1016/j.cellsig.2024.111502 39521028

[86] Bhatia S Arslan E Rodriguez-Hernandez LD Bonin R Wells PG . DNA damage and repair and epigenetic modification in the role of Oxoguanine glycosylase 1 in brain development. Toxicol Sci. (2022) 187:93–111. doi: 10.1093/toxsci/kfac003 35038743

[87] Bishnolia M Yadav P Singh SK Manhar N Rajput S Khurana A . Methyl donor ameliorates CCl4-induced liver fibrosis by inhibiting inflammation, and fibrosis through the downregulation of EGFR and DNMT-1 expression. Food Chem Toxicol. (2025) 196:115230. doi: 10.1016/j.fct.2024.115230 39736447

[88] Downton P Bagnall JS England H Spiller DG Humphreys NE Jackson DA . Overexpression of IκB^+^ modulates NF-κB activation of inflammatory target gene expression. Front Mol Biosci. (2023) 10:1187187. doi: 10.3389/fmolb.2023.1187187 37228587 PMC10203502

[89] Chen J Xu G Xie Z Xie S Luo W Zhong S . GPD2 inhibition impairs coagulation function via ROS/NF-κB/P2Y12 pathway. Cell Mol Biol Lett. (2025) 30:84. doi: 10.1186/s11658-025-00759-x 40682019 PMC12273321

[90] Li LH Huang Y Wang XX Xu CC Wu L He KL . Epigenetic regulators polyphenols in neurodegenerative diseases: a promising intervention strategy. Ann Med. (2026) 58:2634566. doi: 10.1080/07853890.2026.2634566 41918250 PMC13045177

[91] Kaszycki J Kim M . Epigenetic regulation of transcription factors involved in NLRP3 inflammasome and NF-kB signaling pathways. Front Immunol. (2025) 16:1529756. doi: 10.3389/fimmu.2025.1529756 40046056 PMC11879833

[92] Zhang C Zhou Y Hu M Pan Y Chen X Sun Q . PLOD1 promotes the Malignancy of hepatocellular carcinoma by facilitating the NF-κB/IL-6/STAT3-dependent TCA cycle. JHEP Rep. (2025) 7:101329. doi: 10.1016/j.jhepr.2025.101329 40290518 PMC12023786

[93] Yu Z Liu J Chen L Xie J . Role of interleukin-6 in rheumatoid arthritis-associated interstitial lung disease: focus on the JAK/STAT pathway and macrophage polarization. J Inflammation Res. (2025) 18:10953–67. doi: 10.2147/JIR.S530754 40827265 PMC12358124

[94] Wu L Wang X Wang L Li S Chen Q . DNA methylation and demethylation in adipocyte biology: roles of DNMT and TET proteins in metabolic disorders. Front Endocrinol (Lausanne). (2025) 16:1591152. doi: 10.3389/fendo.2025.1591152 40620794 PMC12226276

[95] Sood A Chintalapani S Sharma S Tikoo K . Zebularine ameliorates imiquimod-induced psoriasis by inhibiting oxidative stress and inflammation with concomitant inhibition of NF-κB/MAPK and DNMT1. J Biochem Mol Toxicol. (2025) 39:e70364. doi: 10.1002/jbt.70364 40568777

[96] Peng V Xing X Bando JK Trsan T Di Luccia B Collins PL . Whole-genome profiling of DNA methylation and hydroxymethylation identifies distinct regulatory programs among innate lymphocytes. Nat Immunol. (2022) 23:619–31. doi: 10.1038/s41590-022-01164-8 35332328 PMC8989654

[97] Barbachowska M Arimondo PB . To target or not to target? The role of DNA and histone methylation in bacterial infections. Epigenetics. (2023) 18:2242689. doi: 10.1080/15592294.2023.2242689 37731322 PMC10515666

[98] Liu C Lin L Yao G Fan Y Guo Y . The emerging paradigms of SETD family enzymes as epigenetic regulators of the immune response in inflammatory diseases. Front Immunol. (2026) 17:1725917. doi: 10.3389/fimmu.2026.1725917 41659862 PMC12876154

[99] Zeng Y Jain R Lam M Ahmed M Guo H Xu W . DNA methylation modulated genetic variant effect on gene transcriptional regulation. Genome Biol. (2023) 24:285. doi: 10.1186/s13059-023-03130-5 38066556 PMC10709945

[100] Li X Rao K Chen C Zhang Y Zhou J Meng X . A cell type and state specific gene regulation network inference method for immune regulatory analysis. NPJ Syst Biol Appl. (2025) 11:94. doi: 10.1038/s41540-025-00564-4 40804307 PMC12350830

[101] Martini L Baek SH Lo I Raby BA Silverman EK Weiss ST . Detecting and dissecting signaling crosstalk via the multilayer network integration of signaling and regulatory interactions. Nucleic Acids Res. (2024) 52:e5. doi: 10.1093/nar/gkad1035 37953325 PMC10783515

[102] Nagaraja S Ojeda-Miron L Zhang R Oreskovic E Hock C Hu Y . Epigenetic memory of colitis promotes tumour growth. Nature. (2026) 652:774–83. doi: 10.1038/s41586-026-10258-4 41882356 PMC13083248

[103] León B . Type 2 conventional dendritic cell functional heterogeneity: ontogenically committed or environmentally plastic? Trends Immunol. (2025) 46:104–20. doi: 10.1016/j.it.2024.12.005 39843310 PMC11835539

[104] Xu D Tao X Fan Y Teng Y . Sarcoidosis: molecular mechanisms and therapeutic strategies. Mol BioMed. (2025) 6:6. doi: 10.1186/s43556-025-00244-z 39904950 PMC11794924

[105] Gupta MK Peng H Li Y Xu CJ . The role of DNA methylation in personalized medicine for immune-related diseases. Pharmacol Ther. (2023) 250:108508. doi: 10.1016/j.pharmthera.2023.108508 37567513

[106] Anandakumar H Rauch A Wimmer MI Yarritu A Koch G McParland V . Segmental patterning of microbiota and immune cells in the murine intestinal tract. Gut Microbes. (2024) 16:2398126. doi: 10.1080/19490976.2024.2398126 39254265 PMC11404582

[107] Shen J Du S Zhang Y Li H Liu X Jing J . Bidirectional crosstalk between intestinal epithelium and immune microenvironment in inflammatory bowel disease: mechanisms and therapeutic implications. J Inflammation Res. (2026) 19:538988. doi: 10.2147/JIR.S538988 41835113 PMC12988467

[108] Ma Z Wang Z Cao J Dong Y Chen Y . Regulatory roles of intestinal CD4+ T cells in inflammation and their modulation by the intestinal microbiota. Gut Microbes. (2025) 17:2560019. doi: 10.1080/19490976.2025.2560019 40963293 PMC12452487

[109] Yu Y Qiu Y Zhao M . The role of epigenetics in inflammatory memory. Curr Opin Immunol. (2025) 96:102630. doi: 10.1016/j.coi.2025.102630 40752040

[110] Shi J Xu J Chen YE Li JS Cui Y Shen L . The concurrence of DNA methylation and demethylation is associated with transcription regulation. Nat Commun. (2021) 12:5285. doi: 10.1038/s41467-021-25521-7 34489442 PMC8421433

[111] Musz P Ryś G Fic W Sokal-Dembowska A Jarmakiewicz-Czaja S . Nutrigenomics and epigenetics in the dietary management of inflammatory bowel diseases. Genes (Basel). (2025) 16:1368. doi: 10.3390/genes16111368 41300823 PMC12652803

[112] Gao B Jia K Ya Y . LINC00461 promotes macrophage M1 polarization by inhibiting KLF4 transcription. Immunobiology. (2025) 230:153104. doi: 10.1016/j.imbio.2025.153104 40712323

[113] Migliaccio G Morikka J Del Giudice G . Methylation and transcriptomic profiling reveals short term and long term regulatory responses in polarized macrophages. Comput Struct Biotechnol J. (2024) 25:143–52. doi: 10.1016/j.csbj.2024.08.018 39257962 PMC11385784

[114] Haque M Kaminsky L Abdulqadir R . Lactobacillus acidophilus inhibits the TNF-α-induced increase in intestinal epithelial tight junction permeability via a TLR-2 and PI3K-dependent inhibition of NF-κB activation. Front Immunol. (2024) 15:1348010. doi: 10.3389/fimmu.2024.1348010 39081324 PMC11286488

[115] Nwako JG Patel SD Roach TJ . Enteroendocrine cells regulate intestinal barrier permeability. Am J Physiol Cell Physiol. (2025) 328:C1501–8. doi: 10.1152/ajpcell.01077.2024 40095977 PMC12054964

[116] Górecka A Jura-Półtorak A Koźma EM Szeremeta A Olczyk K Komosińska-Vassev K . Biochemical modulators of tight junctions (TJs): Occludin, claudin-2 and zonulin as biomarkers of intestinal barrier leakage in the diagnosis and assessment of inflammatory bowel disease progression. Molecules. (2024) 29:4577. doi: 10.3390/molecules29194577 39407507 PMC11478261

[117] Jiang Y Chen J Du Y Fan M Shen L . Immune modulation for the patterns of epithelial cell death in inflammatory bowel disease. Int Immunopharmacol. (2025) 154:114462. doi: 10.1016/j.intimp.2025.114462 40186907

[118] O'Guinn ML Handler DA Hsieh JJ Mallicote MU Feliciano K Gayer CP . FXR deletion attenuates intestinal barrier dysfunction in murine acute intestinal inflammation. Am J Physiol Gastrointest Liv Physiol. (2024) 327:G175–87. doi: 10.1152/ajpgi.00063.2024 38860296 PMC11427094

[119] Arras W Oosterlinck B Gassman J . Clinical significance of mucin signatures in inflammatory bowel diseases: a systematic review of their expression patterns, polymorphisms, and post-translational modifications. Inflammation Bowel Dis. (2026) 32:375–89. doi: 10.1093/ibd/izaf293 41284256 PMC12857429

[120] Dehnavi M Galli G García-Estrada C Balaña-Fouce R Giráldez FJ Alonso M . An ovine intestinal organoid-macrophage co-culture model to test the effects of ovine colostrum exosomes on intestinal barrier function and inflammation. Int J Mol Sci. (2025) 26:11406. doi: 10.3390/ijms262311406 41373563 PMC12692019

[121] Uemura I Takahashi-Suzuki N Satoh T . Epigenetic regulation of intestinal innate immunity during differentiation of Caco-2 cells under inflammatory stress. Toxicol Vitro. (2026) 113:106217. doi: 10.1016/j.tiv.2026.106217 41724291

[122] Frazer LC Yamaguchi Y Singh DK Akopyants NS Good M . DNA methylation in necrotizing enterocolitis. Expert Rev Mol Med. (2024) 26:e16. doi: 10.1017/erm.2024.16 38557638 PMC11140546

[123] Wang M Su H Liu J . Metabolic crosstalk between intestinal microbiota and dendritic cells: from homeostasis to inflammation. Acta Biochim Biophys Sin (Shanghai). (2025) 58:156–68. doi: 10.3724/abbs.2025231 41408830 PMC12862611

[124] Walvekar KP Andugulapati SB Chilaka S . A comprehensive analysis of epigenetic mechanisms regulating inflammation-induced TNF-α gene expression by small-molecule inhibitor screening. Mol Biol Rep. (2026) 53:354. doi: 10.1007/s11033-026-11514-6 41632348

[125] Narabayashi H Koma C Nakata K Ikegami M Nakanishi Y Ogihara J . Gut microbiota-dependent adaptor molecule recruits DNA methyltransferase to the TLR4 gene in colonic epithelial cells to suppress inflammatory reactions. Front Mol Biosci. (2022) 9:1005136. doi: 10.3389/fmolb.2022.1005136 36339704 PMC9634067

[126] Ma X Fan W Zhang T Luo L Wang K . The interplay between oxidative stress and epigenetic reprogramming in cancer. Int J Cancer. (2025) 157:2004–18. doi: 10.1002/ijc.70058 40699200

[127] Kharat SS Sharan SK . 5-Hydroxymethylcytosine: a key epigenetic mark in cancer and chemotherapy response. Epigenet Chromatin. (2025) 18:73. doi: 10.1186/s13072-025-00636-z 41243111 PMC12621374

[128] Raynaud CM Jabeen A Ahmed EI Hubrack S Sanchez A Sherif S . MUC2 expression modulates immune infiltration in colorectal cancer. Front Immunol. (2025) 15:1500374. doi: 10.3389/fimmu.2024.1500374 39926604 PMC11802499

[129] An HS Lee J Lee SJ Jeong EA Shin HJ Kim KE . Lipocalin-2 deletion attenuates lipopolysaccharide-induced acute lung inflammation via downregulating chemotaxis-related genes. Biochem Biophys Res Commun. (2023) 652:14–21. doi: 10.1016/j.bbrc.2023.02.029 36806084

[130] Pires S Yang W Frigerio S Louis C Scott C Zhou YL . Innate lymphoid cells activated by the cytokine TL1A link colitis to emergency granulopoiesis and the recruitment of tumor-promoting neutrophils. Immunity. (2026) 59:372–387.e7. doi: 10.1016/j.immuni.2025.12.008 41576959 PMC12981380

[131] Teixeira QE Ferreira DC da Silva AMP Gonçalves LS Pires FR Carrouel F . Aging as a risk factor on the immunoexpression of pro-inflammatory IL-1β, IL-6 and TNF-α cytokines in chronic apical periodontitis lesions. Biol (Basel). (2021) 11:14. doi: 10.3390/biology11010014 35053012 PMC8772771

[132] Zhu Z Turak A Xu N Jenis J Aisa HA . Three new monoterpenes compounds isolated from Seriphidium terrae-albae exerted anti-inflammatory effects through the JAK/STAT and NF-κB signaling pathways. Fitoterapia. (2025) 180:106335. doi: 10.1016/j.fitote.2024.106335 39662632

[133] Xie L Gao F Xu J Xiong W Yin J Sun W . TREM1 enhances macrophage proinflammatory response to LPS by promoting NF-κB activation via an IL-26-mediated JAK/STAT signaling pathway. Iran J Allergy Asthma Immunol. (2026) 25:58–68. doi: 10.18502/ijaai.v25i1.20439 41674173

[134] Williams RO Clanchy FI Huang YS Tseng WY Stone TW . TNFR2 signalling in inflammatory diseases. Best Pract Res Clin Rheumatol. (2024) 38:101941. doi: 10.1016/j.berh.2024.101941 38538489

[135] Xu H Lin S Zhou Z Li D Zhang X Yu M . New genetic and epigenetic insights into the chemokine system: the latest discoveries aiding progression toward precision medicine. Cell Mol Immunol. (2023) 20:739–76. doi: 10.1038/s41423-023-01032-x 37198402 PMC10189238

[136] Damiano OM Stevens AJ Kenwright DN Seddon AR . Chronic inflammation to cancer: the impact of oxidative stress on DNA methylation. Front Biosci (Landmark Ed). (2025) 30:26142. doi: 10.31083/FBL26142 40152377

[137] Margraf A Perretti M . Immune cell plasticity in inflammation: insights into description and regulation of immune cell phenotypes. Cells. (2022) 11:1824. doi: 10.3390/cells11111824 35681519 PMC9180515

[138] Xie K Hunter J Lee A Ahmad G Witting PK Ortiz-Cerda T . The PAD4 inhibitor GSK484 diminishes neutrophil extracellular trap in the colon mucosa but fails to improve inflammatory biomarkers in experimental colitis. Biosci Rep. (2025) 45(6):375–97. doi: 10.1042/BSR20253205 40459411 PMC12236107

[139] Xiao Y Yuan Y Yang Y Liu B Ding Z Luo J . GCH1 reduces LPS-induced alveolar macrophage polarization and inflammation by inhibition of ferroptosis. Inflammation Res. (2023) 72:1941–55. doi: 10.1007/s00011-023-01785-1 37735250

[140] Salloum Z Dauner K Li YF Verma N Valdivieso-González D Almendro-Vedia V . Statin-mediated reduction in mitochondrial cholesterol primes an anti-inflammatory response in macrophages by upregulating Jmjd3. Elife. (2024) 13:e85964. doi: 10.7554/eLife.85964 38602170 PMC11186637

[141] Verstockt B Salas A Sands BE Abraham C Leibovitzh H Neurath MF . IL-12 and IL-23 pathway inhibition in inflammatory bowel disease. Nat Rev Gastroenterol Hepatol. (2023) 20:433–46. doi: 10.1038/s41575-023-00768-1 37069321 PMC10958371

[142] Lv Y Jin YL Zhou Z Liao JB Zhang ZQ Tang LY . The interaction between dendritic cells and T follicular helper cells drives inflammatory bowel disease: a review. Front Immunol. (2026) 17:1725349. doi: 10.3389/fimmu.2026.1725349 41710888 PMC12909234

[143] Wang Y Chen C Yan W Fu Y . Epigenetic modification of m6A methylation: regulatory factors, functions and mechanism in inflammatory bowel disease. Int J Biochem Cell Biol. (2024) 166:106502. doi: 10.1016/j.biocel.2023.106502 38030117

[144] Blot M Léopold V de Beer R Florquin S Butler JM Van't Veer C . The Sirt1 activator SRT1720 mitigates human monocyte activation and improves outcome during gram-negative pneumosepsis in mice. Int J Mol Sci. (2025) 26:9309. doi: 10.3390/ijms26199309 41096578 PMC12525223

[145] Caldwell BA Li L . Epigenetic regulation of innate immune dynamics during inflammation. J Leukoc Biol. (2024) 115:589–606. doi: 10.1093/jleuko/qiae026 38301269 PMC10980576

[146] Dai F Ye S Zhu Y Zhang J . Identification of inflammation-related diagnostic biomarker and molecular subtypes in ulcerative colitis based on machine learning. Dig Dis Sci. (2026) 71:496–508. doi: 10.1007/s10620-025-09312-4 40855234

[147] Cucoreanu C Tigu AB Nistor M Moldovan RC Pralea IE Iacobescu M . Epigenetic and molecular alterations in obesity: linking CRP and DNA methylation to systemic inflammation. Curr Issues Mol Biol. (2024) 46:7430–46. doi: 10.3390/cimb46070441 39057082 PMC11275580

[148] Beckstette M Lu CW Herppich S Diem EC Ntalli A Ochel A . Profiling of epigenetic marker regions in murine ILCs under homeostatic and inflammatory conditions. J Exp Med. (2022) 219:e20210663. doi: 10.1084/jem.20210663 35938981 PMC9386974

[149] Weishaupt K Ackermann J Burger P Chambers D Grimm K Weinkam R . Individual subsets of alternatively-activated macrophages differentially contribute to tissue repair and the resolution of inflammation. J Immunol. (2025) 214:3554–64. doi: 10.1093/jimmun/vkaf164 40795234

[150] Xu C Fu X Qin H Yao K . Traversing the epigenetic landscape: DNA methylation from retina to brain in development and disease. Front Cell Neurosci. (2024) 18:1499719. doi: 10.3389/fncel.2024.1499719 39678047 PMC11637887

[151] Hamdan FH Perez I Kossick K Smith H Edwinson A de Hoyos-Vega JM . Intestinal stem cells from patients with inflammatory bowel disease retain an epigenetic memory of inflammation. (2026) 20:101774. doi: 10.1016/j.jcmgh.2026.101774 PMC13196144 41903684

[152] Caldwell BA Wu Y Wang J Li L . Altered DNA methylation underlies monocyte dysregulation and immune exhaustion memory in sepsis. Cell Rep. (2024) 43:113894. doi: 10.1016/j.celrep.2024.113894 38442017 PMC11654472

[153] Xiong M Sun W . Research progress of probiotics and their protective strategy in the field of inflammatory bowel disease treatment: a review. Med (Balt). (2024) 103:e40401. doi: 10.1097/MD.0000000000040401 39495980 PMC11537665

[154] Bretto E Urpì-Ferreruela M Casanova GR González-Suárez B . The role of gut microbiota in gastrointestinal immune homeostasis and inflammation: implications for inflammatory bowel disease. Biomedicines. (2025) 13:1807. doi: 10.3390/biomedicines13081807 40868062 PMC12383986

[155] Guo J Lou X Gong W Bian J Liao Y Wu Q . The effects of different stress on intestinal mucosal barrier and intestinal microecology were discussed based on three typical animal models. Front Cell Infect Microbiol. (2022) 12:953474. doi: 10.3389/fcimb.2022.953474 36250050 PMC9557054

[156] Ou Q Power R Griffin MD . Revisiting regulatory T cells as modulators of innate immune response and inflammatory diseases. Front Immunol. (2023) 14:1287465. doi: 10.3389/fimmu.2023.1287465 37928540 PMC10623442

[157] Singh A Kaileh M De S Mazan-Mamczarz K Bayarsaihan D Sen R . Transcription factor TFII-I fine tunes innate properties of B lymphocytes. Front Immunol. (2023) 14:1067459. doi: 10.3389/fimmu.2023.1067459 36756127 PMC9900109

[158] Levic DS Niedzwiecki D Kandakatla A Karlovich NS Juneja A Park J . TNF promoter hypomethylation is associated with mucosal inflammation in IBD and anti-TNF response. Gastro Hep Adv. (2024) 3:888–98. doi: 10.1016/j.gastha.2024.06.010 39286616 PMC11402298

[159] Joustra V Li Yim AYF Hageman I Levin E Adams A Satsangi J . Long-term temporal stability of peripheral blood DNA methylation profiles in patients with inflammatory bowel disease. Cell Mol Gastroenterol Hepatol. (2023) 15:869–85. doi: 10.1016/j.jcmgh.2022.12.011 36581079 PMC9972576

[160] Watanabe T Kidoguchi K Kimura S . Treating hematological Malignancies with OR-2100, an orally bioavailable prodrug of decitabine. Cancer Sci. (2025) 116:853–61. doi: 10.1111/cas.16452 39837580 PMC11967254

[161] Brock L Benzien L Lange S Huehns M Runge A Roolf C . KMT2A degradation is observed in decitabine-responsive acute lymphoblastic leukemia cells. Mol Oncol. (2025) 19:1404–21. doi: 10.1002/1878-0261.13792 39754404 PMC12077275

[162] Gawronski M Starczak M Wasilow A Dziaman T Olinski R Gackowski D . Loss of TET2 activity limits the ability of vitamin C to activate DNA demethylation in human HAP1 cells. Epigenet Chromatin. (2025) 18:76. doi: 10.1186/s13072-025-00634-1 41287077 PMC12642257

[163] Huang X Li R Zong M Zhu Y Sun Y Cui H . Targeting NF-κB-inducing kinase (NIK) in the non-canonical pathway: advances in the development of NIK inhibitors. Bioorg Chem. (2025) 167:109247. doi: 10.1016/j.bioorg.2025.109247 41260069

[164] Skudrzyk E Bułdak Ł Machnik G Okopień B . Effect of tofacitinib on the phenotype and activity of Caco-2 cells in a model of inflammatory bowel disease. Exp Ther Med. (2024) 27:152. doi: 10.3892/etm.2024.12440 38476894 PMC10928998

[165] Kumar S Sharma V Yadav S . TLR4 targeting: a promising therapeutic approach across multiple human diseases. Curr Protein Pept Sci. (2025) 26:241–58. doi: 10.2174/0113892037324425241018061548 39722483

[166] Ni X Wang Q Ning Y Liu J Su Q Lv S . Anemoside B4 targets NEK7 to inhibit NLRP3 inflammasome activation and alleviate MSU-induced acute gouty arthritis by modulating the NF-κB signaling pathway. Phytomedicine. (2025) 138:156407. doi: 10.1016/j.phymed.2025.156407 39939033

[167] Fu J Wu H . Structural mechanisms of NLRP3 inflammasome assembly and activation. Annu Rev Immunol. (2023) 41:301–16. doi: 10.1146/annurev-immunol-081022-021207 36750315 PMC10159982

[168] Liu X Li H Yuan M Wan J Guo J Dong X . The epigenetic landscape of tumor-associated macrophages: orchestrating immune evasion in the tumor microenvironment. Cancer Lett. (2025) 632:217972. doi: 10.1016/j.canlet.2025.217972 40780502

[169] Huang HY Zheng XN Tian L . Ontogeny specification and epigenetic regulation of macrophage plasticity. Front Immunol. (2025) 16:1676953. doi: 10.3389/fimmu.2025.1676953 41063992 PMC12500546

[170] Lee JJ Yang L Kotzin JJ Ahimovic D Bale MJ Nigrovic PA . Early transcriptional effects of inflammatory cytokines reveal highly redundant cytokine networks. J Exp Med. (2025) 222:e20241207. doi: 10.1084/jem.20241207 39873673 PMC11865922

[171] Jenkins E Latta C Le HA . Immune cell interactions in development, homeostasis and immunity. J Cell Sci. (2025) 138:jcs264374. doi: 10.1242/jcs.264374 41031606

[172] Li J Yang H Zhu M Zhang P Liu Y Niu Y . Unlocking the therapeutic potential of the STING signaling pathway in anti-tumor treatment. Clin Exp Med. (2025) 25:290. doi: 10.1007/s10238-025-01838-1 40794212 PMC12343656

